# Long-term inhibition of lysosomal glucocerebrosidase activity promotes GPX4 stability and inhibits ferroptosis in dopaminergic neurons

**DOI:** 10.1038/s41419-026-08833-8

**Published:** 2026-06-08

**Authors:** Marie-Amandine Bonte, Flore Gouel, Aurélie Jonneaux, Karim Belarbi, David Devos, Jean-Christophe Devedjian

**Affiliations:** 1 Département de Pharmacologie Médicale, Université de Lille, LilNCog - Lille Neuroscience & Cognition, Equipe TREAT, INSERM UMRS_1172, CHU Lille, LICEND COEN Center, Lille, France; 2 Université de Lille, UFR 3S, Département de Pharmacologie, Lille, France; 3 Université Du Littoral Côte D’Opale, 1, Place de l’Yser, Dunkerque, Cedex 1 France

**Keywords:** Chaperone-mediated autophagy, Cell death, Cellular neuroscience, Cell death in the nervous system

## Abstract

An increasing number of studies indicate that ferroptosis, a lethal pathway initiated by excessive iron-dependent lipid peroxidation, and pivotal to the survival of dopaminergic neurons and the progression of Parkinson’s disease (PD), may be regulated by the lysosomal pathway. Mutation and loss of function of the lysosomal enzyme, glucocerebrosidase, induce the accumulation of glycosphingolipids and alterations in lysosome activity, which have been associated with a higher risk of developing PD. Our present study showed that transient inhibition of glucocerebrosidase activity had a positive effect on lipid peroxidation and ferroptosis. In a dopaminergic cell line (LUHMES cells), it was shown that a 10-day inhibition of glucocerebrosidase activity using conduritol-beta-epoxide (CBE) specifically impeded susceptibility to RSL3-induced ferroptosis, but not to several other inducers of cell death. CBE impaired the lysosomal pathway, modified lipid membrane composition by reducing ether-linked phospholipids in phosphatidylethanolamines, and promoted an increase in glutathione peroxidase 4 (GPX4) protein levels. This phenomenon was transient and disappeared after 20 days of glucocerebrosidase inhibition, suggesting that the cells have the capacity to return to their basal homeostasis. Most of the current compounds acting on GPX4 promote its degradation, thus information on drugs leading to GPX4 stability is key in order to protect neurons against excessive lipid peroxidation occurring in neurodegenerative diseases.

## Introduction

Programmed and regulated cell death varies and is a complex process. This highly controlled process involves sequences of events allowing multicellular organisms to eliminate dysfunctional cells. When they are deregulated, several diseases such as cancer and neurodegenerative diseases may occur. For a long time, the main regulated cell death was considered to be excessive apoptosis, followed by autophagy-dependent death, but it is only more recently that the different types of regulated death have been described and shown to be relevant in the context of neurodegenerative diseases.

We have demonstrated that ferroptosis is pivotal in several models and situations of dopaminergic neuronal death related to Parkinson’s disease (PD) [1]. Ferroptosis is a lethal pathway promoted by excessive lipid peroxidation. The involvement of iron in ferroptosis is essential for lipid peroxidation and regulation of iron import, export, or storage by ferritin modifies susceptibility to ferroptosis. Among the other important players in ferroptosis, anti-oxidant enzymes and glutathione peroxidase 4 (GPX4) in particular, combat lipid peroxidation while acyl-CoA synthetase long-chain family member 4 (ACSL4) is an essential component for ferroptosis execution by shaping cellular lipid composition [2].

Rather than a specific pathway, it is the interplay between the different cell death effectors that appears to play a central role in cell fate. Autophagy has already been described as coinciding with apoptosis and recent data suggest that autophagy may also interact with the execution of ferroptosis [3, 4]. In response to classical ferroptosis activators, an increased autophagy flux is observed in various cells [4]. In addition, certain types of selective autophagy (i.e., ferritinophagy, lipophagy) appear to promote ferroptosis due to increased lipid peroxidation [4]. The molecular and metabolic basis of autophagy-dependent ferroptosis remain largely unknown and we question whether autophagy could regulate membrane lipid composition and subsequent ferroptosis in neurons.

Lysosomal glucocerebrosidase is an enzyme involved in the hydrolysis of glucosylceramides into glucose and ceramide. Glycosphingolipids are relatively abundant components of neuronal membranes, particularly in lipid rafts. They also appear to be involved in several biological processes such as neuronal maturation, or endocytic and lysosomal pathways [5–8]. Lysosomal glucocerebrosidase has been studied extensively in Gaucher disease, a lysosomal storage disorder due to a loss-of-function mutation present in both alleles of the *GBA* gene encoding lysosomal glucocerebrosidase [9–12]. In addition, the presence of a mutation in one allele of *GBA* has been associated with a higher risk of developing PD [13]. A reduction in glucocerebrosidase activity has been observed in several brain regions, notably in the substantia nigra, in PD patients with a *GBA* mutation [14]. It was also shown that PD patients without a *GBA* mutation have a decrease in glucocerebrosidase enzyme activity, but to a lesser extent [14].

The current study investigated the effects of long-term inhibition of lysosomal glucocerebrosidase activity and its effect on cell survival against neurotoxins in a dopaminergic cell line (LUHMES). Our results show that long-term inhibition of lysosomal glucocerebrosidase using conduritol-beta-epoxide (CBE) promotes strong and specific protection against ferroptosis. This inhibition modifies lipid membrane composition and impairs the lysosomal pathway. Neuroprotection against ferroptosis could result directly from increased GPX4 stability.

## Results

### Long-term impairment of lysosomal glucocerebrosidase activity can be modeled in a dopaminergic-like neuron model

**Fig. 1:**
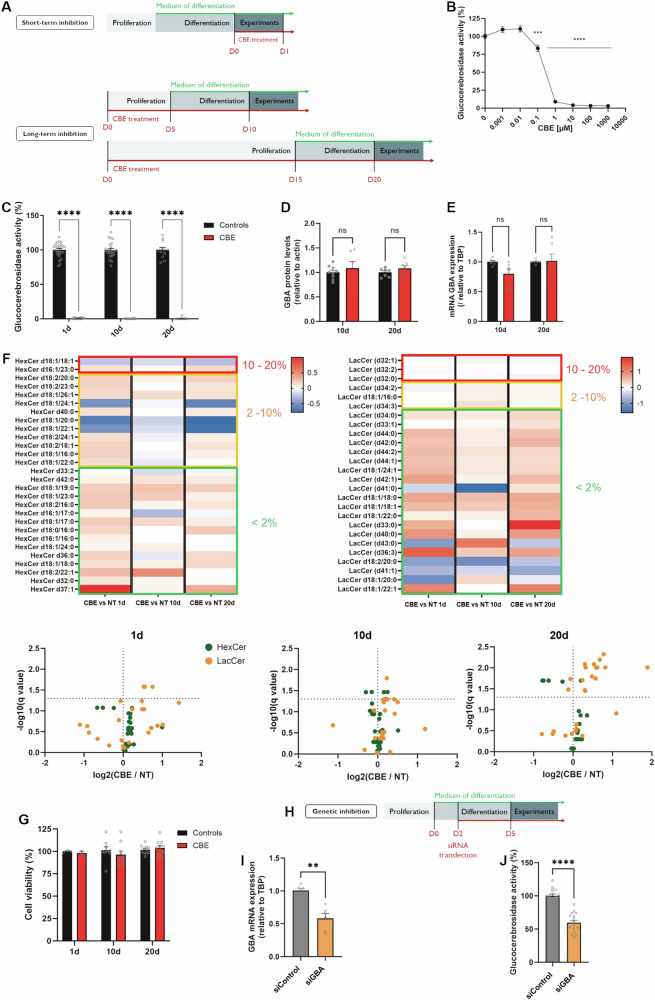
Characterization of lysosomal glucocerebrosidase deficiency models in dopaminergic-like neurons. Timelines of pharmacological inhibition of lysosomal glucocerebrosidase activity. **A** Schema of experimental design of short- and long-term inhibition of lysosomal glucocerebrosidase activity. **B** Measurement of lysosomal glucocerebrosidase activity with a range of concentrations of conduritol-beta-epoxide (CBE) for 24 h. **C** Measurement of lysosomal glucocerebrosidase activity after treatment with 10 µM CBE for 1 day, 10 days, and 20 days *vs*. controls. **D** Protein and **E** mRNA expression of glucocerebrosidase after long-term inhibition *vs*. controls. **F** Heatmaps and volcano plots comparing the expression of monohexosylceramides (HexCer) or lactosylceramides (LacCer) in CBE cells treated for 1 day, 10 days, or 20 days *vs*. controls. For the heatmaps, glycosphingolipids were ranked from the most expressed to the least expressed, and subdivided into three classes representing the percentage expression (between 10 and 20%, between 2 and 10%, or <2%). For the volcano plots, HexCer are represented in green and LacCer in orange. **G** Cell viability after treatment with 10 µM CBE for 1 day, 10 days, and 20 days *vs*. controls. **H** Timeline of genetic inhibition of lysosomal glucocerebrosidase. **I** Glucocerebrosidase mRNA expression of siGBA *vs*. siControl. **J** Measurement of lysosomal glucocerebrosidase activity in the siGBA condition *vs*. the siControl condition. All experiments were carried out in triplicate and the results are expressed as the mean ± standard error of the mean. **p < 0.01; ***p < 0.001; ****p < 0.0001.

Pharmacological and genetic inhibition of lysosomal glucocerebrosidase were carried out to investigate the impact of lysosomal glucocerebrosidase deficiency on LUHMES cell susceptibility to cell death. LUHMES cells were exposed for 1 day, 10 days, or 20 days to a specific inhibitor of lysosomal glucocerebrosidase activity, CBE (Fig. 1A). The effective concentration of CBE determined after 24 h indicated that 10 µM induced total inhibition of glucocerebrosidase activity (Fig. 1B) (IC50 of CBE ~ 9 uM). Total inhibition was also effective at 10 days and 20 days (Fig. 1C) without affecting glucocerebrosidase protein and mRNA levels (Fig. 1D, E). Inhibition of lysosomal glucocerebrosidase activity did not affect cell viability (Fig. 1G). An increase in monohexosylceramides and lactosylceramides, which are part of glycosphingolipids, was also observed in CBE-treated cells compared to controls at all time points (Fig. 1F), showing CBE efficiency to inhibit glucocerebrosidase activity reinforcing data already published [15]. The genetic approach used siRNA directed against *GBA*. A 40% reduction in *GBA* mRNA was obtained, associated with a 50% decrease in enzymatic activity (Fig. 1I, J). Thus, different cellular models of short- or long-term lysosomal glucocerebrosidase deficiency were established to study their impact on the susceptibility of dopaminergic-like neurons to cell death.

### 10-day inhibition of lysosomal glucocerebrosidase activity changes cellular sensitivity to ferroptosis

**Fig. 2:**
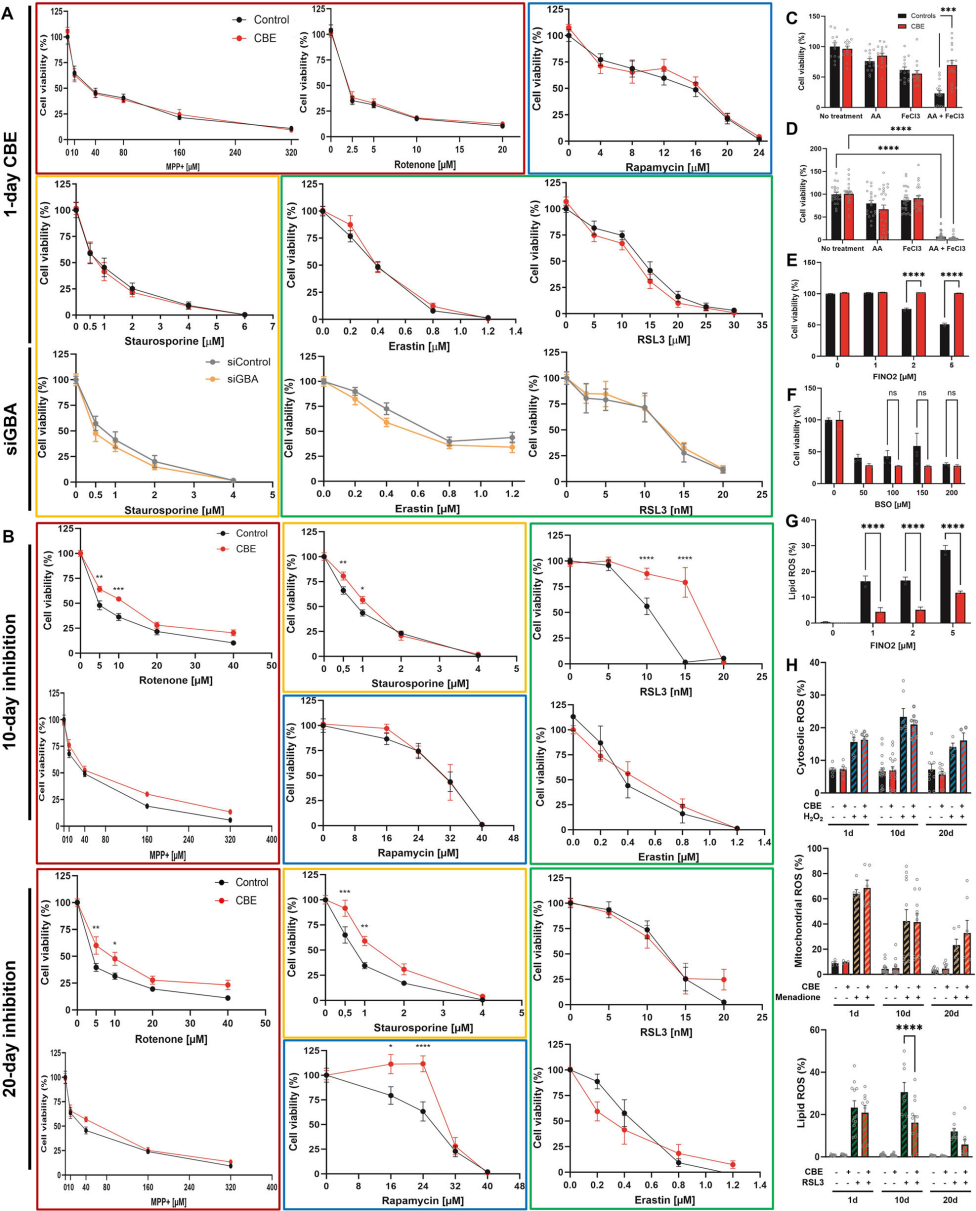
10-day inhibition of lysosomal glucocerebrosidase activity changes the sensitivity to ferroptosis. **A, B** Cell viability of short-term (**A)** and long-term (**B)** glucocerebrosidase deficiency models treated with a range of concentrations of various cell stimuli to induce cell death, induced by mitochondrial alterations (red box), apoptosis (yellow box), autophagy (blue box), and ferroptosis (green box). **C**, **D** Cell viability after co-treatment with arachidonic acid and FeCl_3_ in untreated cells or CBE-treated cells for 10 days (**C)** or 20 days (**D)**. **E**, **F** Cell viability after a range of concentrations of FINO2 (**E)** or BSO (**F)** in untreated cells or CBE-treated cells for 10 days. **G** Quantification of ROS lipids induced with 1, 2 and 5 µM of FINO2 on untreated cells or CBE-treated cells for 10 days. **H** Assessment of cytosolic, mitochondrial, and lipidic reactive oxygen species (ROS) after treatment with 10 µM CBE for 1 day, 10 days, or 20 days. All experiments were performed in triplicate and the results are expressed as the mean ± standard error of the mean. *p < 0.05; **p < 0.01; ***p < 0.001; ****p < 0.0001.

Apoptosis and autophagy are well described cell death processes in dopaminergic neurodegeneration. It has also been shown that dopaminergic neurons are sensitive to changes in mitochondrial integrity induced by MPP+ or rotenone, and more recently, to ferroptosis [1]. We studied the impact of lysosomal glucocerebrosidase deficiency on LUHMES cells treated with different cell death inducers. Short-term inhibition with CBE did not alter the sensitivity of LUHMES cells to the inducers used (Fig. 2A). Long-term inhibition of glucocerebrosidase activity slightly reduced the sensitivity of LUHMES cells to staurosporine and rotenone, but no difference was observed with MPP + . Cells treated with CBE for 20 days were protected against rapamycin (Fig. 2B). Cells treated with CBE for 10 days were strongly protected against RSL3-induced ferroptosis compared to controls and this neuroprotection effect was abolished in 20-day CBE cells. However, no difference was observed for erastin, another inducer of ferroptosis, suggesting a specific ferroptosis pathway (Fig. 2B). To avoid an experimental artefact between RSL3 and CBE, we used another pro-ferroptotic condition consisting of a combination of arachidonic acid (AA) and ferric chloride was also tested [16]. Similarly, 10-day CBE cells were protected against this co-treatment (Fig. 2C) while no difference was observed with 20-day CBE cells compared to controls (Fig. 2D). To confirm our results we used two other ferroptosis inducers: FINO2, another GPX4 inhibitor like RSL3, and BSO which induce a depletion of glutathione and which can, at least partially, act similarly to erastin. Significant neuroprotection was observed against FINO2 (Fig. 2E), while no difference was observed against BSO for 10-day CBE (Fig. 2F). These results confirm our previous observations suggesting that 10-day CBE show neuroprotection against ferroptosis induced specifically by GPX4 inhibitors. Lipid peroxidation is a hallmark of ferroptosis. Oxidative stress analysis was performed by measuring cytosolic, mitochondrial, or lipid ROS induced by H_2_O_2_, menadione, or RSL3, respectively. No difference in the production of cytosolic and mitochondrial ROS was observed. However, there was a significant reduction in RSL3-induced lipid peroxidation in 10-day CBE cells but not 20-day CBE cells (Fig. 2H). Furthermore, a significant reduction in FINO2-induced lipid peroxidation in 10-day CBE was also observed (Fig. 2G). Taken together, these results suggest that CBE-induced specific, but transient, protection against ferroptosis in LUHMES cells.

### 10-day inhibition of lysosomal glucocerebrosidase activity changes the lipid composition of membranes

**Fig. 3:**
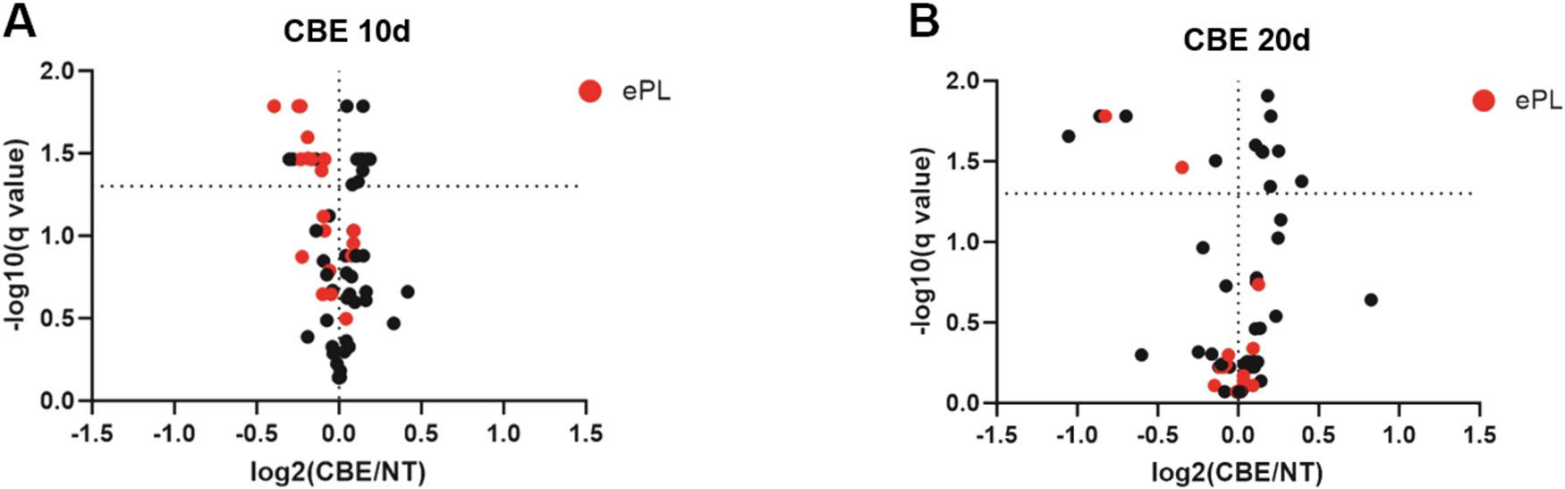
Sensitivity to ferroptosis is mediated by ether-linked phosphatidylethanolamines (PE). Volcano plots of the changes in expression to PE, with ePL colored in red for 10-day (**A)** or 20-day conduritol-beta-epoxide-treated cells (**B)** vs. controls (four replicates were used per condition).

Ether-linked phospholids (ePL) are phospholipids where the sn-1 tail group is bound to the glycerol backbone through an ether linkage (rather than an ester bond in diacyl phospholipids). There are two subclasses of ePL: alkyl-ether phospholipids and vinyl-ether phospholipids, depending on whether the ether-linked carbohydrate chain is saturated or contains a double bond. ePL are more likely to contain polyunsaturated fatty acid (PUFA) than their diacyl counterparts [17]. Recently, studies have shown that ePL are implicated in ferroptosis susceptibility and their reduction can protect cells from ferroptosis [18, 19]. Furthermore, reduced expression of ACSL4 in LUHMES cells, a pro-ferroptotic factor that participates in PUFA enrichment of membranes, downregulated ePL levels, suggesting that ACSL4 could modulate the synthesis of ePL [20]. Furthermore, ether-linked phosphatidylethanolamine (PE) phospholipids may be key executioners of ferroptosis as they are likely to contain PUFA and tend to localize on the inner leaflet of the plasma membrane where they are presumably exposed to oxidative conditions, while phosphatidylcholine species (PC) appear less sensitive to lipid peroxidation due to their localization in the outer leaflet of the plasma membrane [17]. Lipid profiling of PE molecular species in 10-day or 20-day CBE cells was performed to determine the ePL levels. A majority of ePL in PE species (red dots) was significantly decreased in 10-day CBE cells (Fig. 3A), while only two ePL in PE species were observed in 20-day CBE cells (Fig. 3B). The ePL in PC species tended to increase in CBE-treated cells compared to controls, but this was not significant (Supplementary Fig. 1A, B). These results reinforce the link between susceptibility and ePL in PE species to ferroptosis and suggest the involvement of the lysosomal pathway in modulating these lipids.

### 10-day inhibition of lysosomal glucocerebrosidase modulates the expression of key players in ferroptosis

Ferroptosis is regulated by several mechanisms mainly involving the metabolism of lipids and iron [21]. To better understand the neuroprotective effect of glucocerebrosidase inhibition against ferroptosis, the mRNA and protein expression of several players involved in this regulation were assessed. For lipid metabolism, we focused on GPX4, the antioxidant enzyme that detoxifies peroxidized lipids into their alcohol analog, and on ACSL4. For iron metabolism, we focused on ferritin, an iron storage protein, on the transferrin receptor involved in the import of iron into the cell, as well as heme oxygenase, an enzyme involved in the release of iron from heme.

**Fig. 4:**
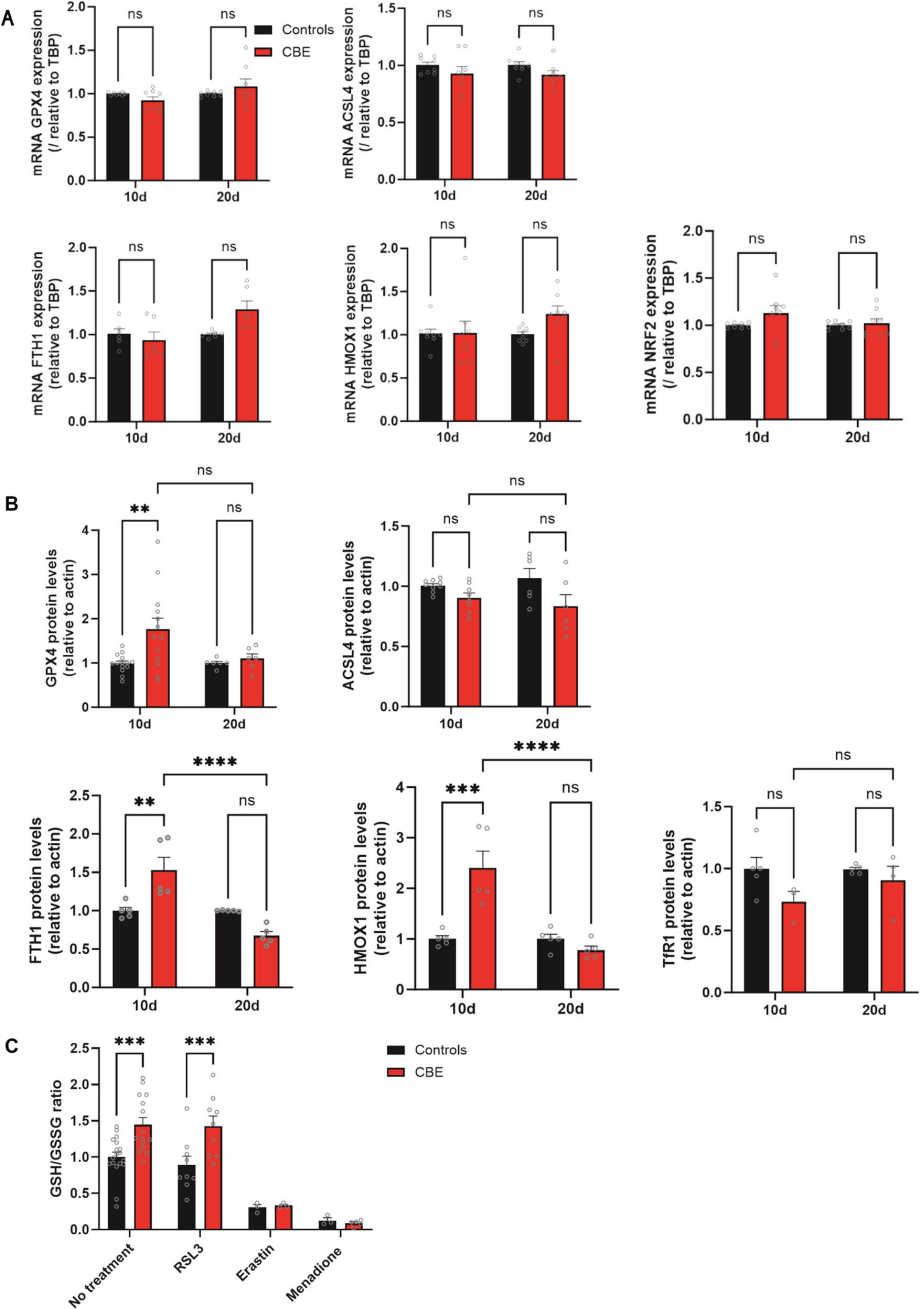
10-day inhibition of lysosomal glucocerebrosidase changes the expression of key players in ferroptosis. **A** mRNA levels of GPX4, ACSL4, FTH1, HMOX1, and NRF2 in conduritol-beta-epoxide (CBE)-treated cells vs. controls. **B** Protein levels of GPX4, ACSL4, FTH1, HMOX1, and TfR1 in CBE-treated cells vs. controls. **C** GSH/GSSG ratio of CBE-treated cells vs. controls. All experiments were carried out in triplicate and the results are expressed as the mean ± standard error of the mean. **p < 0.01; ***p < 0.001; ****p < 0.0001.

For all markers, mRNA levels did not change between CBE-treated cells and controls (Fig. 4A). There was a significant increase in GPX4 protein levels in 10-day CBE cells, but no difference was found in 20-day CBE cells compared to controls (Fig. 4B). No difference was also found in ACSL4 protein levels. A recent study showed that alpha-synuclein can shape membrane lipids and determine ferroptosis sensitivity [20] (20). No difference was observed in alpha-synuclein protein levels (in supplementary data).

Concerning iron metabolism, there was a significant increase in ferritin and heme oxygenase protein levels in 10-day CBE cells, whereas no difference was observed in 20-day CBE cells (Fig. 4B). There was no difference in transferrin receptor protein expression (Fig. 4B). Finally, we also measured the mRNA levels of transcription factor NRF2, involved in the regulation of many genes that regulate ferroptosis. Again, no difference was found in CBE-treated cells compared to controls (Fig. 4A). Taken together, these results indicate an increase in protein expression of several players involved in the regulation of ferroptosis in 10-day CBE cells, while no difference was found at the transcriptional level. It can be hypothesized that protein deregulation results from a defect in protein degradation by the lysosomal pathway. Moreover, the activity of GPX4 requires reduced GSH as a cofactor giving oxidized GSSG. When the GSH/GSSG ratio was measured in 10-day CBE cells, an increase in the ratio was observed meaning that the cofactor of GPX4 was more readily available, allowing the enzyme to be active (Fig. 4C). Erastin and menadione were used as positive controls to induce a depletion of GSH, and to generate ROS, respectively and strongly decrease the GSH/GSSG ratio (Fig. 4C). In view of the results, showing a change in lipid composition and expression of several ferroptosis players in 10-day CBE cells, we focused on these conditions for subsequent experiments.

### 10-day inhibition of lysosomal glucocerebrosidase activity disrupts the autophagic flow

**Fig. 5:**
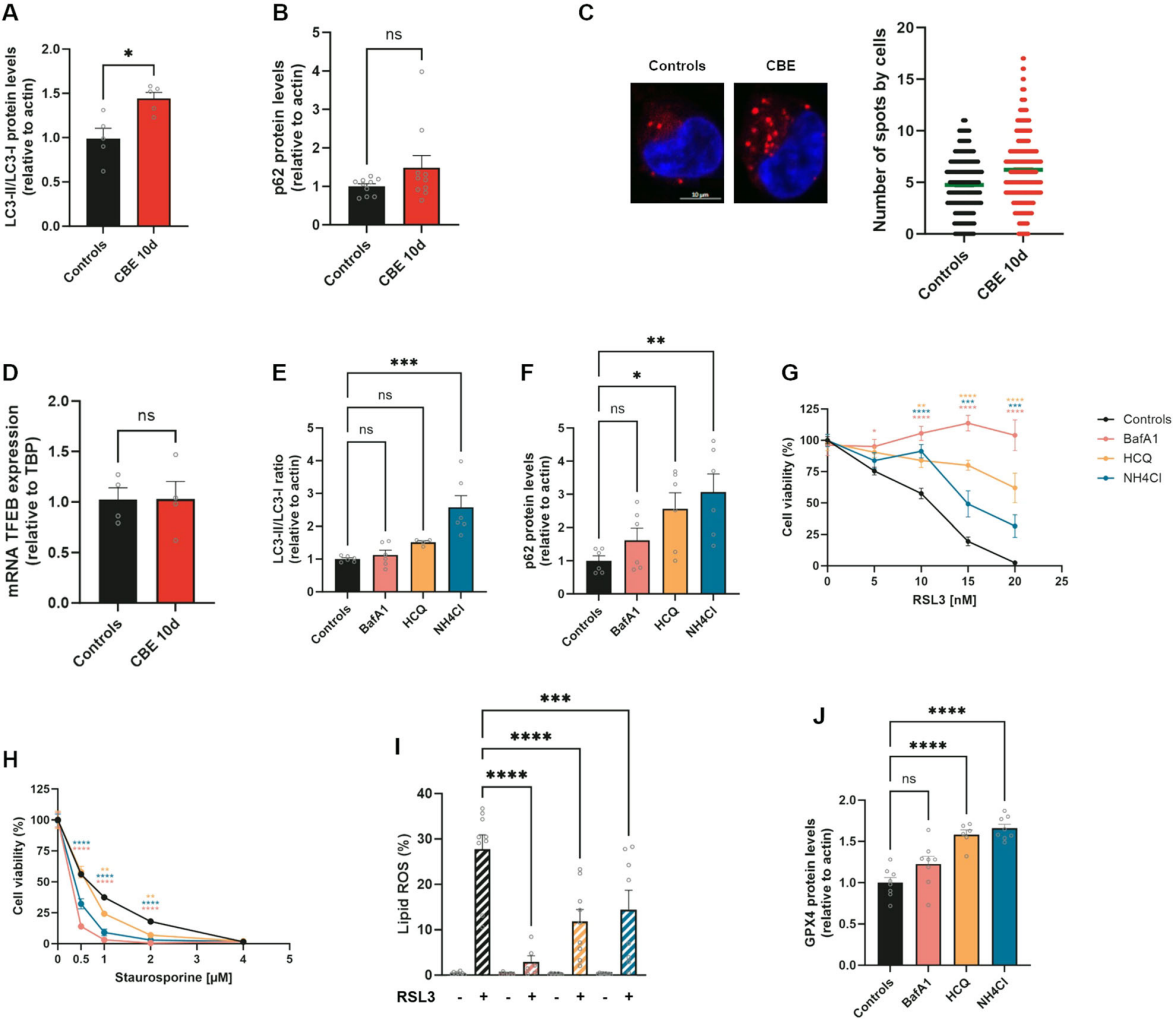
10-day inhibition of lysosomal glucocerebrosidase transiently alters the autophagic flow. **A**, **B** Analysis of LC3b **(A)** and p62 (**B)** protein levels in cells treated with CBE for 10 days *vs*. controls. **C** Representative images and lysosomal quantification. **D** TFEB mRNA expression in cells treated with CBE for 10 days *vs*. controls. **E, F** Western blot analysis of LC3b (**E)** and p62 (**F)** proteins levels in cells treated with autophagy inhibitors for 7 h *vs*. controls. **G**, **H** Cell viability relative to RLS3 (**G)** or staurosporine (**H)** after treatment with autophagy inhibitors for 1 h in untreated cells. **I** Quantification of RSL3-initiated lipid reactive oxygen species after treatment with autophagy inhibitors for 1 h. **J** Western analysis of GPX4 proteins levels in cell treated with autophagy inhibitors for 7 h. All experiments were performed in triplicate and the results are expressed as the mean ± standard error of the mean. *p < 0.05; **p < 0.01; ***p < 0.001; ****p < 0.0001.

Autophagy is a cellular mechanism involving the degradation of intracellular components by the lysosomal pathway. Microtubule-associated protein 1 light chain 3 (LC3) and p62/SQSTM1 (i.e., sequestosome) are markers used to monitor autophagy. To determine whether the modulation of the proteins levels observed in 10-day CBE cells result from a disruption of the lysosomal pathway, these autophagic markers were measured. A significant increase in the LC3-II/LC3-I ratio was observed while no difference was found in the p62 protein levels (Fig. 5A and B).

These increases in autophagy markers were correlated with an increase in the number of lysosomes observed in 10-day CBE cells (Fig. 5C). To go further, the mRNA levels of TFEB, a transcription factor involved in the regulation of lysosome biogenesis, were also measured. No difference was found between 10-day CBE cells and controls. These results suggest that the observed protein level changes in CBE-treated cells were not due to an increase in lysosome synthesis but rather to an alteration in autophagic flux. To test this hypothesis, common inhibitors of autophagy flux were used such as BafA1 or HCQ, which inhibit the late stage of autophagy, and NH_4_Cl, responsible for the basification of lysosomes [22]. A significant increase in the LC3-II/LC3-I ratio was observed for the NH_4_Cl condition (Fig. 5E) and an increase in p62 protein levels for HCQ and NH_4_Cl conditions (Fig. 5F). These results are consistent with impaired autophagic flux. To assess the impact of altered autophagic flux on ferroptosis sensitivity, cells were pre-treated with the inhibitors and then exposed to RSL3. Neuroprotection against RSL3 (Fig. 5G) and a reduction in RSL3-induced lipid peroxidation was observed for all autophagy inhibitors (Fig. 5I). This effect was specific to ferroptosis because cells pretreated with autophagy inhibitors were more sensitive to apoptosis (Fig. 5H). Finally, a significant increase in GPX4 levels was observed for HCQ and NH_4_Cl compared to controls (Fig. 5J). Collectively, these observations are consistent with the results obtained for the 10-day CBE condition and show that the lysosomal pathway is involved in the regulation of ferroptosis in LUHMES cells.

### Increased half-life of GPX4 may prevent death by ferroptosis

We next hypothesized that the neuroprotection observed against RSL3-induced ferroptosis in 10-days CBE cells primarily results from an increase in GPX4 expression. To assess this hypothesis, GPX4 silencing was induced to evaluate whether its deficiency was sufficient to sensitize CBE-treated LUHMES cells to ferroptosis.

**Fig. 6:**
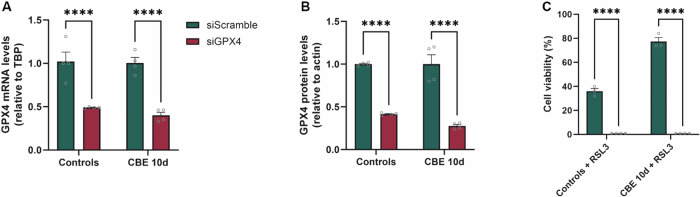
Increased half-life of GPX4 may protect dopaminergic-like neurons from ferroptosis. **A** GPX4 mRNA expression and **B** protein levels after transfection with siGPX4 *vs*. siControl in CBE-treated or control cells. **C** Cell viability of CBE-treated or control cells transfected with siGPX4 or siControl after treatment with 10 nM RSL3 for 24 h. All experiments were performed in triplicate and the results are expressed as the mean ± standard error of the mean. **p < 0.01; ****p < 0.0001.

A 50% and 60% decrease in GPX4 was observed in untreated or 10-day CBE cells transfected with siGPX4, respectively, compared to cells transfected with siRNA control (Fig. 6A). A decrease in GPX4 protein levels was also observed in cells transfected with siGPX4 (Fig. 6B). Finally, both untreated and CBE-treated cells transfected with siGPX4 exhibited complete mortality, suggesting that increased GPX4 half-life is key in 10-day CBE induced neuroprotection against excessive lipid peroxidation and subsequent ferroptosis (Fig. 6C).

## Discussion

This study shows that 10-day inhibition of glucocerebrosidase activity in a dopaminergic cell line could modulate susceptibility to ferroptosis by modifying lipid composition and altering protein degradation of key players like GPX4 involved in the regulation of ferroptosis. However, this phenomenon is transient, suggesting that the cells have the capacity to return to a state of basal homeostasis, highlighting the complexity of cellular mechanisms.

First identified as an original cell death pathway, an increasing number of studies indicate that ferroptosis may occur by sharing common effectors with other types of regulated cell death like autophagy [23, 24] and lysosomal cell death [25, 26]. Mutations in *GBA* inducing a loss of function of a lysosomal enzyme have been associated with a higher risk of developing PD. Thus, we studied the interaction between lysosomal storage disorder induced by glucocerebrosidase inhibition and ferroptosis in dopaminergic-like neurons.

CBE is a well-known and effective inhibitor of lysosomal glucocerebrosidase [27]. Given that inhibition of a single lysosomal enzyme takes time to alter a system such as the lysosomal pathway, the inhibition of glucocerebrosidase for several days appeared more relevant in LUHMES cells. Our study shows that up to 20 days of treatment, CBE did not impact LUHMES cell viability in basal conditions. CBE treatment inhibited lysosomal glucocerebrosidase activity without impacting its mRNA or protein levels and increased glycosphingolipid species. Thus, long-term impairment of glucocerebrosidase activity can be modeled in this dopaminergic-like neuron model. Long-term inhibition of glucocerebrosidase activity using CBE, for 14 or 29 days, has already been achieved in another dopaminergic neuronal model derived from human induced pluripotent stem cells [15].

LUHMES cells are sensitive to many toxins allowing the study of several cell death pathways. Short- or long-term glucocerebrosidase deficiency in LUHMES cells did not alter sensitivity to several cell death inducers. CBE cells were protected against rapamycin, probably due to disruption of lysosomal trafficking. More importantly, 10-day CBE cells were transiently and strongly protected against RSL3-induced ferroptosis while no protection against erastin was observed. The neuroprotective effect was transient as it was not observed at 20 days suggesting that the cells are capable of returning to a state of basal homeostasis and showing that it is interesting to vary study times, as observations may differ. Erastin and RSL3 promote the reduction of antioxidant activity by inhibiting the cystine-glutamate transmembrane antiporter causing cysteine and glutathione depletion or by directly inactivating GPX4, respectively. We also used two other ferroptosis inducers, FINO2, a GPX4 inhibitor, and BSO, a glutamate-cystine ligase inhibitor, with similar mechanisms of action to RSL3 and erastin, respectively. The 10-day CBE were protected against FINO2 but not BSO, suggesting that 10-day CBE are protected against GPX4 inhibitor-induced ferroptosis, meaning that GPX4 may be linked to the observed phenomenon.

To discard an artefactual interaction between CBE and RSL3, another pro-ferroptotic condition consisting of a combination of AA and ferric chloride was used. It has previously been demonstrated that by enriching the membrane with AA, LUHMES cells are more sensitive to GPX4-dependent lipid peroxidation [16]. 10-day CBE cells were protected against this co-treatment while no difference was observed after 20 days. Furthermore, analysis of oxidative stress supported these results since a significant reduction in RSL3-induced lipid peroxidation was observed in 10-day CBE cells. In the SOD1 transgenic mouse model of amyotrophic lateral sclerosis, a study showed that CBE preserves motor neurons [28].

As glucocerebrosidase inhibition seemed to specifically affect lipid composition and peroxidation, we assessed the expression of the main players involved in the regulation of ferroptosis. A transient upregulation of GPX4, HMOX1, and FTH1 proteins levels was observed in 10-day CBE cells but not 20-day CBE cells compared to controls. In contrast to these results, excessive activation of HMOX1 has been shown to enhance ferroptosis while pharmacological inhibition or silencing of HMOX1 confers resistance to ferroptosis [29–31]. However, HMOX1 may also act in a cytoprotective manner, probably depending on the level of activation. The protective effect of HMOX1 is attributed to its antioxidant activity whereas its toxic effect is due to increased generation of ferrous iron, which stimulates the Fenton reaction. Thus, over-regulation of HMOX1 could be cytotoxic, while moderate regulation could be cytoprotective [32, 33]. Iron release and FTH1 degradation have already been shown to be regulated through ferritinophagy, a selective form of autophagy, which can release sufficient iron to trigger ferroptosis. In PC12 cells, FTH1 overexpression impairs ferritinophagy, suppressing ferroptosis-induced cell death [34]. Furthermore, inhibition of lysosomal activity or silencing of nuclear receptor coactivator 4 (a cargo receptor recruiting FTH1 to autophagosomes for lysosomal degradation) suppresses ferroptosis [24, 23, 26]. Interestingly, erastin but not RSL3 seems to promote ferritinophagy in HeLa cells [35]. Therefore, CBE could be more efficient against RSL3 since the mechanism of action of RSL3 appears to be independent of the ferritin degradation process. Although the direct inactivation of GPX4 by RSL3 is uncertain [36], the role of GPX4 is central in scavenging lipid peroxidation. Subsequent to GBA inhibition, GPX4 is more abundant in the cells and its higher activity translated into an increased GSH/GSSG ratio. However, we observed a non-significant decrease in the GSH/GSSG ratio after exposure to RSL3. GPX4, being inhibited by RSL3, is not expected to use GSH as a co-factor for lipid detoxification. The decrease in this ratio could result from residual activity of GPX4 or other GSH-utilizing enzymes such as glutharedoxins.

This is certainly one of the main effects of CBE that makes cells more resistant to RSL3-induced ferroptosis. As with FTH1, GPX4 regulation by autophagy is well documented [37]. For example, acid sphingomyelinase, a key enzyme in sphingolipid metabolism, mediates activation of autophagy and induces GPX4 degradation. Conversely, inhibition of this enzyme reduces GPX4 degradation by autophagy [22].

We then determined whether protein upregulation following GBA inhibition resulted from a defect in their degradation by the lysosomal pathway. In 10-day CBE cells, a disruption of the lysosomal pathway was measured by an increase in the LC3-II/LC3-I ratio, an increase in the number of lysosomes, and no difference in lysosome biogenesis. Impaired lysosomal activity was observed after long-term treatment with CBE in another dopaminergic neurons model. This lysosomal alteration was measured by increased levels of lysosomal associated membrane protein 1 and TFEB nuclear translocation as well as the number and size of lysosomes [15]. A reduction in lysosome catalytic activity was also found in this model using radioactive glycosphingolipids [15]. These results are consistent with our observations on lysosomal pathway impairment. To complete the demonstration that impaired autophagic flux may be responsible for resistance to ferroptosis, potent autophagic flux inhibitors were used. Short treatment was sufficient to perturb autophagic flux and protect against ferroptosis. It was noticeable that the inhibitor with the strongest effect on neuroprotection and lipid peroxidation, BafA1, seemed to have the least effect on GPX4 protein levels; a significant increase in GPX4 protein was observed for the HCQ and NH_4_Cl conditions. Finally, GPX4 silencing restored RSL3-induced ferroptosis sensitivity in 10-day CBE cells showing that the neuroprotection observed could result directly from increased GPX4 expression.

In recent years, the number of studies on ferroptosis has increased dramatically, revealing a surprising degree of complexity. Concerning the interaction between cell death pathways, it is a real challenge to define the threshold or checkpoints associated with pro-survival and pro-death autophagy in ferroptosis. Loss of GBA function has been associated with a higher risk of developing PD, probably due to long-term consequences on glycosphingolipids and lysosomal activity. This study shows that transient inhibition of GBA activity could have a positive effect on lipid peroxidation. If sustained for a relatively short length of time, by altering the lysosomal pathway, an inhibitor like CBE modified lipid composition and increased GPX4 half-life. Various small molecule compounds can affect GPX4 expression, mainly leading to its degradation [38]. It is therefore important to better understand the different pathways leading to GPX4 degradation in order to develop specific GPX4 activators to protect neurons against excessive lipid peroxidation and subsequent ferroptosis.

## Materials and methods

### Pharmacological treatments

The pharmacological compounds used for the different experiments are shown in Table 1.

**Table 1:** Pharmacological compounds used in the experiments.

Pharmacological compound	Conditions of treatment	Supplier
Ammonium chloride (NH_4_Cl)	20 mM added 1 h before other treatments	Sigma-Aldrich, Saint-Louis, Missouri, USA
Arachidonic acid	20 µM added 2 h before FeCl_3_ and left for 48 h	Sigma-Aldrich
Bafilomycine A1	10 nM added 1 h before other treatments	Selleckchem, Cologne, Germany
Buthioninesulfoximine (BSO)	Range of concentrations for 24h	Sigma-Aldrich
Conduritol-beta-epoxide	10 µM for 1, 10, or 20 days	Millipore, Burlington, Massachussets, USA
Erastin	Range of concentrations for 24 h	Sigma-Aldrich
Ferric chloride (FeCl_3_)	20 µM FeCl_3_ for 48 h and left for 48 h	Sigma-Aldrich
FINO_2_	Viability: range of concentrations for 24h; lipidic ROS: range of concentrations for 6h	Selleckchem
H_2_O_2_	Cytosolic ROS: 100 µM for 30 min	VWR Chemicals, Radnor, Pennsylvania, USA
Hydroxychloroquine	10 µM added 1 h before other treatments	Selleckchem
Menadione	Mitochondrial ROS: 100 µM for 1 h; glutathione assay: 10 µM for 6 h	Sigma-Aldrich
MPP+	Range of concentrations for 24 h	Sigma-Aldrich
Rapamycin	Range of concentrations for 24 h	Selleckchem
Rotenone	Range of concentrations for 24 h	Sigma-Aldrich
RSL3	Viability: range of concentrations for 24 h; lipidic ROS: 30 nM for 6 h	Selleckchem
Staurosporine	Range of concentrations for 24 h	Sigma-Aldrich

### LUHMES cell culture

The human dopaminergic neuronal LUHMES (Lund Human Mesencephalic) cell line is a subclone of a mesencephalic cell line originally derived from ventral mesencephalic tissue of an 8-week-old female human embryo [39]. LUHMES cells were generated and characterized by the laboratory of Prof. Marcel Leist (University of Konstanz, Germany) and are commercially available through ATCC (CRL-2927). The cell line has a normal karyotype and has been authenticated by short tandem repeat (STR) profiling. LUHMES cells were kindly provided to our laboratory by Prof. Marcel Leist’s group and were cultured according to established protocols. Testing of LUHMES cells for mycoplasma contamination was performed using standard detection assays. The LUHMES cells were maintained at 37 °C in a humidified 95% air, 5% CO_2_ atmosphere. Proliferative cells were cultured in Advanced DMEM/F12 (Gibco, Waltham, Massachusetts, USA) supplemented with L-glutamine (2 mM; Gibco), N2 (1X, Gibco), and recombinant human basic fibroblast growth factor (bFGF, 40 ng/ml; Miltenyi, Bergish Gladbach, Germany). For dopaminergic neuron differentiation, the proliferative medium was replaced by differentiation medium containing Advanced DMEM/F12 supplemented with 2 mM L-glutamine, N2 (1X), 1 mM dibutyryl cyclic-AMP sodium salt (cAMP; Sigma-Aldrich), 1 µg/ml tetracycline (Sigma Aldrich), and 2 ng/ml glial cell line-derived neurotrophic factor (GDNF; bio-techne, Minneapolis, Minnesota, USA). Two days later, the cells were harvested with 0.05% tryspin-EDTA and re-plated at a density of 4 ×10^4^ cells/well in a 96-well plate, 2.5 ×10^5^ cells/well in a 24-well plate, and 1 ×10^6^ cells/well in a 6-well plate. Cells were left to fully differentiate for another 3 days, until day 5. The flasks or plates were coated with a solution containing 50 µg/ml polo-L-ornithine (Sigma-Aldrich) and 1 µg/ml fibronectin (Sigma-Aldrich) diluted in sterile water for 24 h at 37°C. The flasks or plates were rinsed twice with sterile water and stored at 4°C until use.

### siRNA transfection

siRNA was used to inhibit GPX4 or GBA mRNA expression. For the transfection, a first solution containing lipofectamine (Lipofectamine^TM^ RNAiMAX Transfection Reagent; ThermoFisher Scientific, Waltham, Massachusetts, USA), and a second solution containing Opti-MEM^TM^ Reduced Serum Medium (ThermoFisher Scientific) and siGPX4 (sc-44465, SantaCruz), siScramble (Control siRNA-A, sc-37007; Santa Cruz, Dallas, Texas), or siGBA (L-006366-00-0005; Dharmacon, Horizon, Lafayette, Colorado, USA) and siControl (D-001810-10, Dharmacon, Horizon) were prepared separately. After 5 min incubation, the two solutions were mixed at an equal volume, incubated for 15 min and added to the plates: 10 µl, 100 µl, and 300 µl for 96-well, 24-well, and 6-well plates, respectively. Medium containing the cells was added to a total volume of 100 µl, 1 ml, and 2 ml for 96-well, 24-well, and 6-well plates, respectively. The final concentration was 30 nM for siGPX4 and 20 nM for siGBA. Pre-differentiated cells at day 2 were seeded directly into the mix and differentiation continued for a further 3 days to allow experiments on differentiated LUHMES cells at day 5.

### Real-time PCR

Total RNA was extracted according to the manufacturer’s instructions (RNAeasy mini kit; Qiagen, Hilden, Germany). The quantity of RNA was measured by Nanodrop (ThermoFisher). A DNase step was performed on 2 µg of RNA (Kit Roche). Reverse transcription was performed using Superscript III reverse transcriptase enzyme and random primers (ThermoFisher). Finally, real-time PCR was carried out with a Lightcycler FastStart DNA Master SYBR Green I kit (Roche). The primers are listed in Supplementary Table 1. The housekeeping gene control was TATA-binding protein (TBP). Threshold cycles were assessed for genes and expression levels were reported versus TBP.

### Western-blotting

For protein extraction, cells were rinsed with PBS, harvested with 0.05% trypsin-EDTA and centrifuged at 300 g and 4°C for 5 min. The pellet was lysed in RIPA buffer containing 1% proteases and phosphatase inhibitors. Lysis was completed by a 20 s sonication step with pulses of 1 s every 0.5 s at 20% amplitude. The lysates were centrifuged at 10,000 rpm for 10 min at 4°C to remove cell debris. Proteins were quantified using the BCA protein assay kit (ThermoFisher Scientific). The lysates were mixed with RIPA and 4X loading buffer to obtain a protein concentration of 1 µg/µl and samples were denatured by heating at 95°C for 5 min. Samples were loaded onto 4–20% Tris-glycine gels (BIO-RAD, Hercules, California, USA). Transfer to nitrocellulose membranes was performed with a Power Blotter Station (Invitrogen) for 8 min at 2.5 mA. Membranes were blocked in 5% non-fat dry milk or bovine serum albumin lyophilized powder in TBS-Tween 20 0,1% (TBS-T). The membranes were then incubated with the primary antibody overnight at 4°C (antibodies are listed in Supplementary Table 2). After 3 ×5 min of washing with TBS-T and 3 ×5 min with TBS, membranes were incubated for 1 h at room temperature (RT) with secondary antibodies conjugated to horseradish peroxidase and detected by enhanced chemiluminescence with Amersham ECL detection reagents. Chemiluminescence signals were visualized with Amersham ImageQuant^TM^ 800 (GE Healthcare Life, Milwaukee, Wisconsin, USA) and quantification of the signals was performed using ImageQuant^TM^ TL analysis software with protein quantification normalized to β-actin signals. Western-blot images have been added as Fig. 2 **to** 7 as Supplementary Data. All methods were performed in accordance with the relevant guidelines and regulations.

### Enzymatic activity of lysosomal glucocerebrosidase

Enzyme activity was determined according to the protocol published by Trapero et al. [40]. Briefly, the cells were rinsed once with warmed PBS. The cells were then lysed in a vol/vol mixture of acetate buffer (200 mM, pH 4) and Hank’s balanced salt solution (Sigma-Aldrich) for 30 min at 37°C. An equal volume of 4-methylumbelliferone (5 mM in acetate buffer), in the presence or not of 100 µM CBE (Millipore) was then added and incubated for 1 h at 37°C. The reaction was stopped with an equal volume of glycine buffer (100 mM, pH 10.6). After mixing, a volume of each sample was transferred to a black plate for the measurement of fluorescence with a Mithras LB940 plate reader (Berthold), excitation 365 nm and emission 455 nm.

### Cell viability assay

At day 5 of differentiation, the LUHMES cells were treated with the different cell death inducers and cell viability was assessed 24- or 48-h post-treatment. The cells were incubated with 100 µg/ml resazurin sodium salt (Sigma-Aldrich) for 1 h 45 min at 37°C. After incubation, fluorescence was measured at an excitation/emission wavelength of 540/600 nm using a Mithras LB940 plate reader (Berthold). Viability of the treated cells was reported as a percentage relative to untreated control cells.

### Flow cytometry

At day 5 of differentiation, the LUHMES cells were treated with oxidative stress inducers. The supernatant and cells were collected after tryspinization and centrifuged at 300 g and 4 °C for 5 min. After elimination of the supernatant, the pellets were resuspended with the fluorescent probes diluted in PBS and incubated for 15 min at 37 °C. The final concentrations used for the probes were: CM-H2DCFDA (50 nM), C-11-BODIPY 581/591 (1 µM), and MitoSOX (1 µM) (all purchased from ThermoFisher). These were used to determine intracellular reactive oxygen species (ROS), lipid peroxidation, and mitochondrial ROS, respectively. A total of 15 000 cells was acquired using cytoFLEX (Beckman Coulter, California, USA) and quantification was performed with Kaluza Analysis software (Beckman). Flow cytometry histograms gated have been added as an overlay in Figures 8 to 11 as Supplementary Data.

### Lysosome imaging

On day 2, cells were seeded in a µ-slide 8-well (80826, ibidi) at a density of 125 000 cells/well in a volume of 300 µl until day 5 of differentiation. A total of 150 µl was removed and replaced with medium containing Lysotracker Deep Red (lysosome staining; 50 nM) and Hoescht (nucleus staining; 15 µg/ml), and the cells were incubated for 30 min at 37 °C. Cells were rinsed twice by removing 150 µl of medium and adding 150 µl of heated PBS, and once by removing all medium and replacing with 300 µl of PBS. Live cell images of lysosomes were captured using a confocal microscope (Zeiss LSM 710; Airyscan, Oberkochen, Germany) equipped with a 63X oil immersion objective. The number of lysosomes was counted manually.

### Glutathione assay

Detection and quantification of reduced glutathione (GSH) and oxidized glutathione (GSSG) were carried out using a luminometric kit (GSH/GSSG-Glo assay) following the manufacturer’s instructions (Promega, Madison, Wisconsin, USA). Briefly, after treatment, the medium was removed from the cells. The cells were lysed with 50 µl of total glutathione or oxidized glutathione reagent and shaken for 5 min at RT. Luciferin generation reagent (50 µl) was added to the wells and incubated for 30 min at RT. Luciferin detection reagent (100 µl) was then added and incubated for 15 min at RT. Finally, 150 µl per well was transferred to a plate for luminescence measurement using a microplate reader (Tecan Spark 10 M, Männedorf, Zurich, Switzerland). The ratio of glutathione content was calculated by using the relative light units recorded by the microplate reader. GSH and GSSG levels were calculated according the manufacturer’s guidelines and are represented as the GSH/GSSG ratio.

### Lipid extraction

Lipids were extracted from the cells according to the method of Folch et al. [41]. Briefly, 3 ×10^6^ cells were homogenized with 2 ml of NaCl solution in water (0.73%). Lipids were extracted with 10 ml of CHCl_3_/CH_3_OH (2:1, v/v), and vortexed for 1 min. The mixture was centrifuged at 3000 rpm for 3 min. The upper phase was discarded and the lower phase collected through a protein interface using a Pasteur pipette. After evaporation, the lipid extract (lower phase) was re-dissolved in 200 ml of CHCl_3_/CH_3_OH (2:1, v/v) and stored under nitrogen at -20°C until further analysis.

### Analysis of phospholipid molecular species

A total of 10 µl of internal standards mixture containing 320 µg/ml PC (14:0/14:0) and 160 µg/ml PE (14:0/14:0) were added to 200 µl lipid extract. The process of identification and quantification of phospholipids was performed using a Thermo UltiMate^TM^ 3000 coupled to an Orbitrap Fusion Tribrid mass spectrometer equipped with an EASY-MAX NG ion source (H-ESI; Thermo Scientific). Separation of phospholipid classes was achieved under HILIC conditions using a Kinetex Hilic 100 ×2.1 mm, 1.7 µm column (Phenomenex, Torrance, California, USA), with a flow of 0.5 ml/min. The mobile phase consisted of: (i) CH_3_CN/H_2_O (96/4, v/v) containing 10 mM ammonium acetate; and (ii) CH_3_CN/H_2_O (50/50, v/v) containing 10 mM ammonium acetate. The injection volume was 10 µl and the column was maintained at 50°C. Phospholipids were detected by high resolution mass spectrometry, and H-ESI source parameters were optimized and set as follows: ion transfer tube temperature 285 °C, vaporizer temperature 370°C, sheath gas flow rate 35 au, sweep gas 1 au, and auxiliary gas flow rate 25 au. Positive and negative ions were monitored alternatively by switching polarity with a static spray voltage at 3500 V and 2800 V in positive and negative, respectively. Mass spectra (MS) in full scan mode were obtained using an Orbitrap mass analyzer with the normal mass range and a target resolution of 240 000 (FWHM at m/z 200), on a mass range to charge ratio m/z from 200–1600 using a Quadrupole isolation on a normal mass range. All MS data were recorded using a maximum injection time of 100 ms, automated gain AGC target (%) at 112.5, RF lens (%) at 50 and one microscan. An intensity threshold filter of 1 ×10^3^ counts was applied. For MS/MS analysis, the data-dependent mode was used for the characterization of phospholipid species. Precursor isolation was performed in the Quadrupole analyzer with an isolation width of m/z 1.6. Higher-energy collisional dissociation was employed for the fragmentation of phospholipids with optimized stepped collision energy of 27%. The linear ion trap was used to acquire spectra for fragment ions in data-dependent mode. The AGC target was set to 2 ×10^4^ with a maximum injection time of 50 ms. All MS and MS/MS data were acquired in the profile mode. Orbitrap fusion was controlled by Xcalibur 4.1 software. Data for high accuracy and the information collected from fragmentation spectra, with the help of LipidSearch software (Thermo) and the LIPID MAPS database, were used for phospholipid identification.

### Statistical analysis

Quantitative data are shown as the mean ± standard error of the mean. The number of biological replicates for each experiment is indicated in the figure legends. Depending on the type of analyses, significant differences were evaluated using the Mann-Whitney test, one-way, or two-way ANOVA with Bonferroni’s multiple comparisons test, and were considered significant at *p* < 0.05. All statistical analysis were performed using Prism 9 GraphPad software.

## Supplementary information

Sensitivity to ferroptosis is not mediated by ether-linked phosphatidylcholines.

10-day CBE did not modify the alpha-synuclein protein levels.

Supplemental Table 1. Primer sequences used for real-time PCR experiments.

Supplemental Table 2. Antibodies used for Western-blotting experiments.

Supplemental data for Western blot analysis

Supplemental data for flow cytometry analysis

Legends for supplemental figures and legends

## Data Availability

All data will be available on request from the first author at bontemarieamandine@gmail.com.
