# Supplementary material for: Long-term inhibition of lysosomal glucocerebrosidase activity promotes GPX4 stability and inhibits ferroptosis in dopaminergic neurons
Source: Cell Death Dis. 2026 Jun 8;17(1):717. doi: 10.1038/s41419-026-08833-8 (PMC13470012; doi:10.1038/s41419-026-08833-8)
Supplement: Supplementary file 1 [file 41419_2026_8833_MOESM1_ESM.pdf]

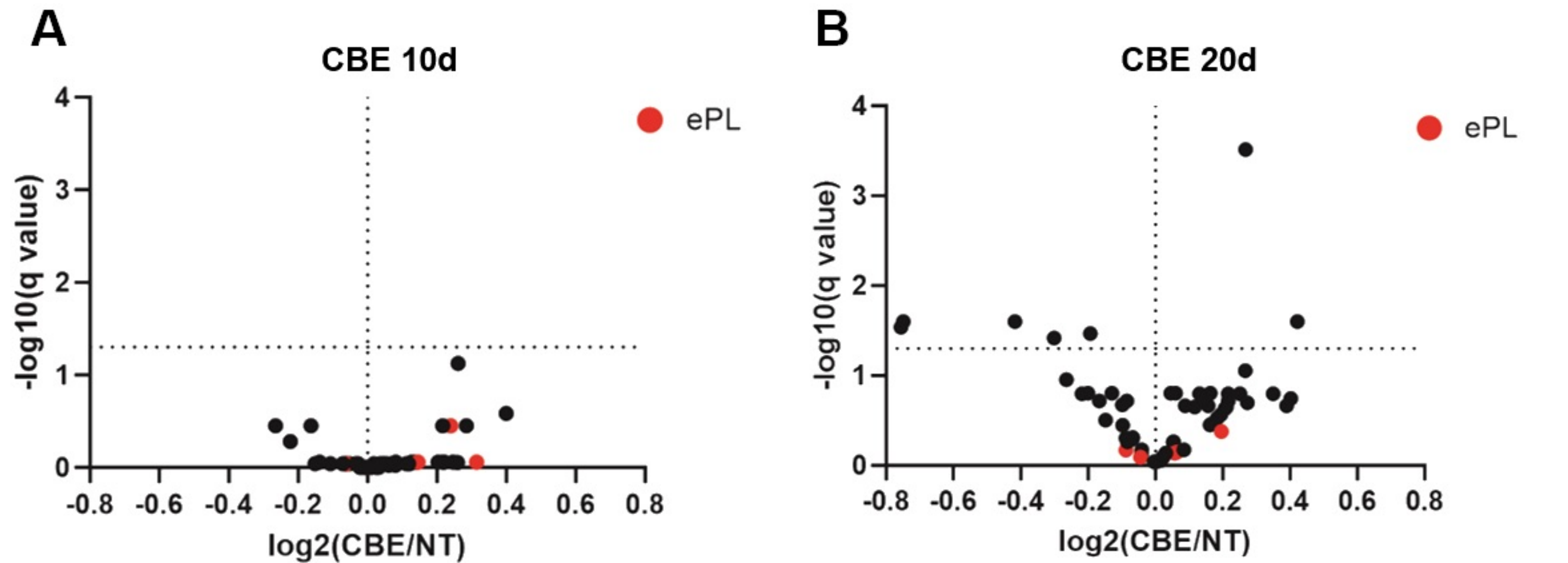
A
CBE 10d
ePL
-log10(q value)
log2(CBE/NT)
4
3
2
1
0
-0.8
-0.6
-0.4
-0.2
0.0
0.2
0.4
0.6
0.8
B
CBE 20d
ePL
-log10(q value)
log2(CBE/NT)
