# Supplementary material for: Long-term inhibition of lysosomal glucocerebrosidase activity promotes GPX4 stability and inhibits ferroptosis in dopaminergic neurons
Source: Cell Death Dis. 2026 Jun 8;17(1):717. doi: 10.1038/s41419-026-08833-8 (PMC13470012; doi:10.1038/s41419-026-08833-8)
Supplement: Supplementary file 2 [file 41419_2026_8833_MOESM2_ESM.pdf]

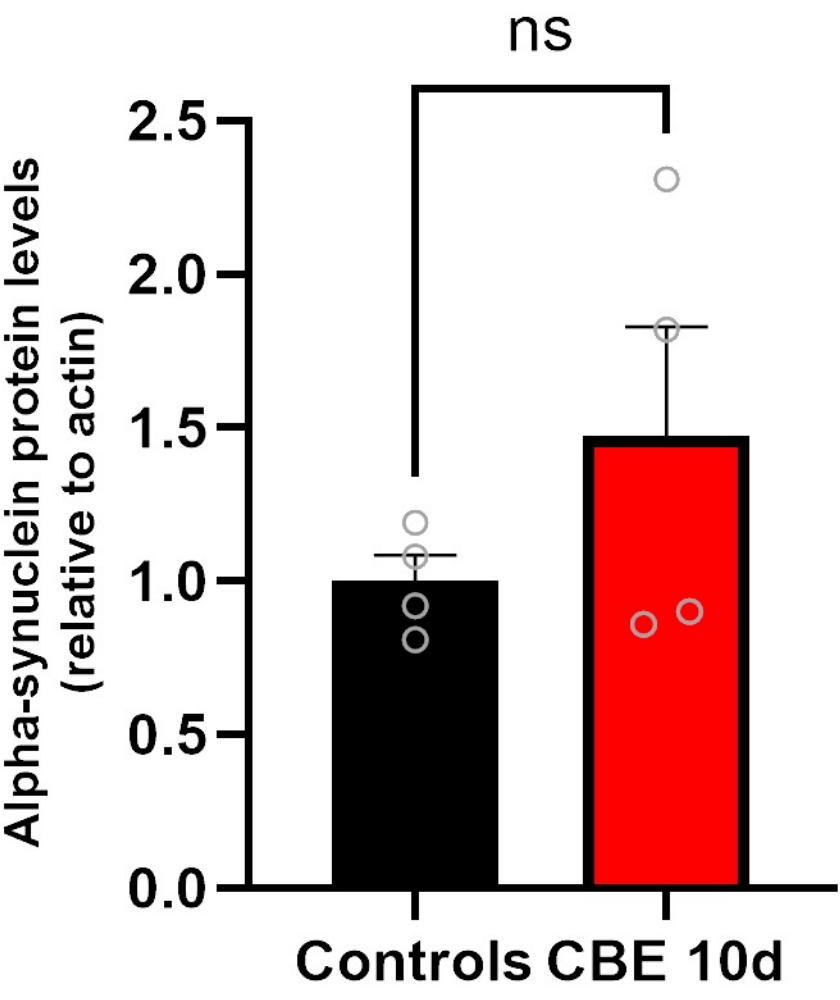
ns
2.5
2.0
1.5
1.0
0.5
0.0
Alpha-synuclein protein levels
(relative to actin)
Controls
CBE 10d
