# Supplementary material for: Long-term inhibition of lysosomal glucocerebrosidase activity promotes GPX4 stability and inhibits ferroptosis in dopaminergic neurons
Source: Cell Death Dis. 2026 Jun 8;17(1):717. doi: 10.1038/s41419-026-08833-8 (PMC13470012; doi:10.1038/s41419-026-08833-8)
Supplement: Supplementary file 3 [file 41419_2026_8833_MOESM3_ESM.pdf]

**Target**	**Forward primer** (5'>3')	**Reverse primer** (5'>3')
ACSL4	TGTACTGTACTGAAGCCCACACTT	TTCATCTCTTGGACTTTGCTCA
FTH1	CGCCAGAACTACCACCAGGAC	GCCACATCATCGCGGTCAA
GBA	CTTCTGCTGGGCTGTTGAGT	TACTGTTGGCGAGGGTAGGA
GPX4	CGGCGAACTCTTTGATCTCT	TTCCCGTGTAACCAGTTCG
HMOX1	AAGACTGCGTTCCTGCTCAAC	AAAGCCCTACAGCAACTGTCG
NRF2	TTTGGGAATGTGGGCAAC	GAGACAGGTGAATTTCTCCCAAT
TBP	CACACGCCAAGAAACAGTGA	CGGCTGTTTAACTTCGCTTC

Supplemental Table 1. Primer sequences used for real-time PCR experiments.
