# Supplementary material for: Long-term inhibition of lysosomal glucocerebrosidase activity promotes GPX4 stability and inhibits ferroptosis in dopaminergic neurons
Source: Cell Death Dis. 2026 Jun 8;17(1):717. doi: 10.1038/s41419-026-08833-8 (PMC13470012; doi:10.1038/s41419-026-08833-8)
Supplement: Supplementary file 4 [file 41419_2026_8833_MOESM4_ESM.pdf]

**Target**	**Reference**
ACSL4	ab155282; abcam, Cambridge, UK
β-actin	sc-47778; Santa Cruz
Ferritin heavy chain	4393S; cell signaling, Danvers, Massachussetts, USA
GPX4	ab125066; abcam
HMOX1	A1346; ABclonal, Woburn, USA
LC3	NB100-2200; Novus Biological, Colorado, USA
p62	H00008878-M01; Novus Biological
Transferrin receptor	13-6800; Thermofisher

Supplemental Table 2. Antibodies used for Western-blotting experiments.
