# Supplementary material for: Long-term inhibition of lysosomal glucocerebrosidase activity promotes GPX4 stability and inhibits ferroptosis in dopaminergic neurons
Source: Cell Death Dis. 2026 Jun 8;17(1):717. doi: 10.1038/s41419-026-08833-8 (PMC13470012; doi:10.1038/s41419-026-08833-8)
Supplement: Supplementary file 5 [file 41419_2026_8833_MOESM5_ESM.pdf]

SUPPLEMENTAL DATA (WESTERN BLOTS)

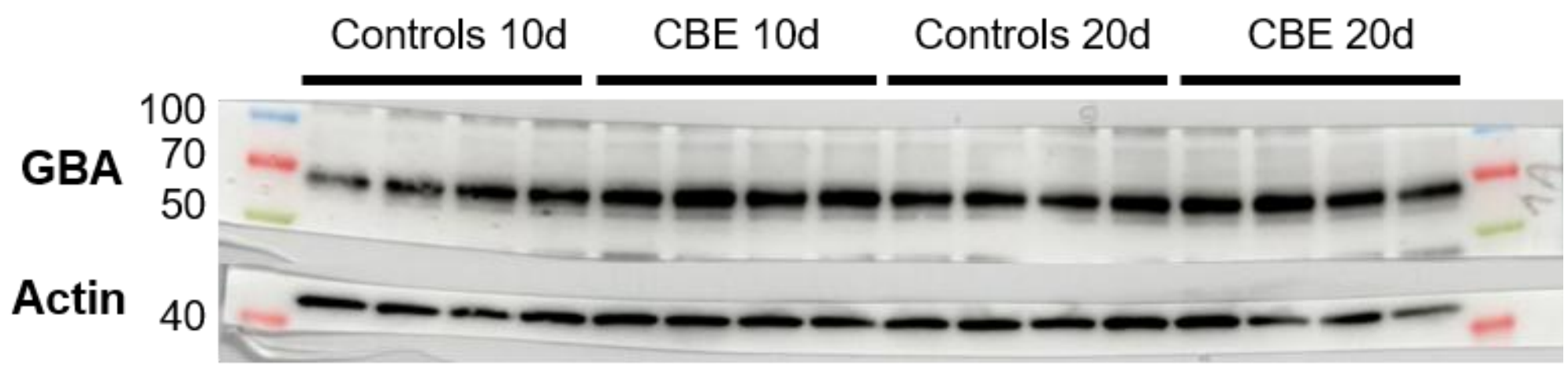

Supplemental Figure 3. Western-blots of glucocerebrosidase in CBE-treated cells *vs*. controls.

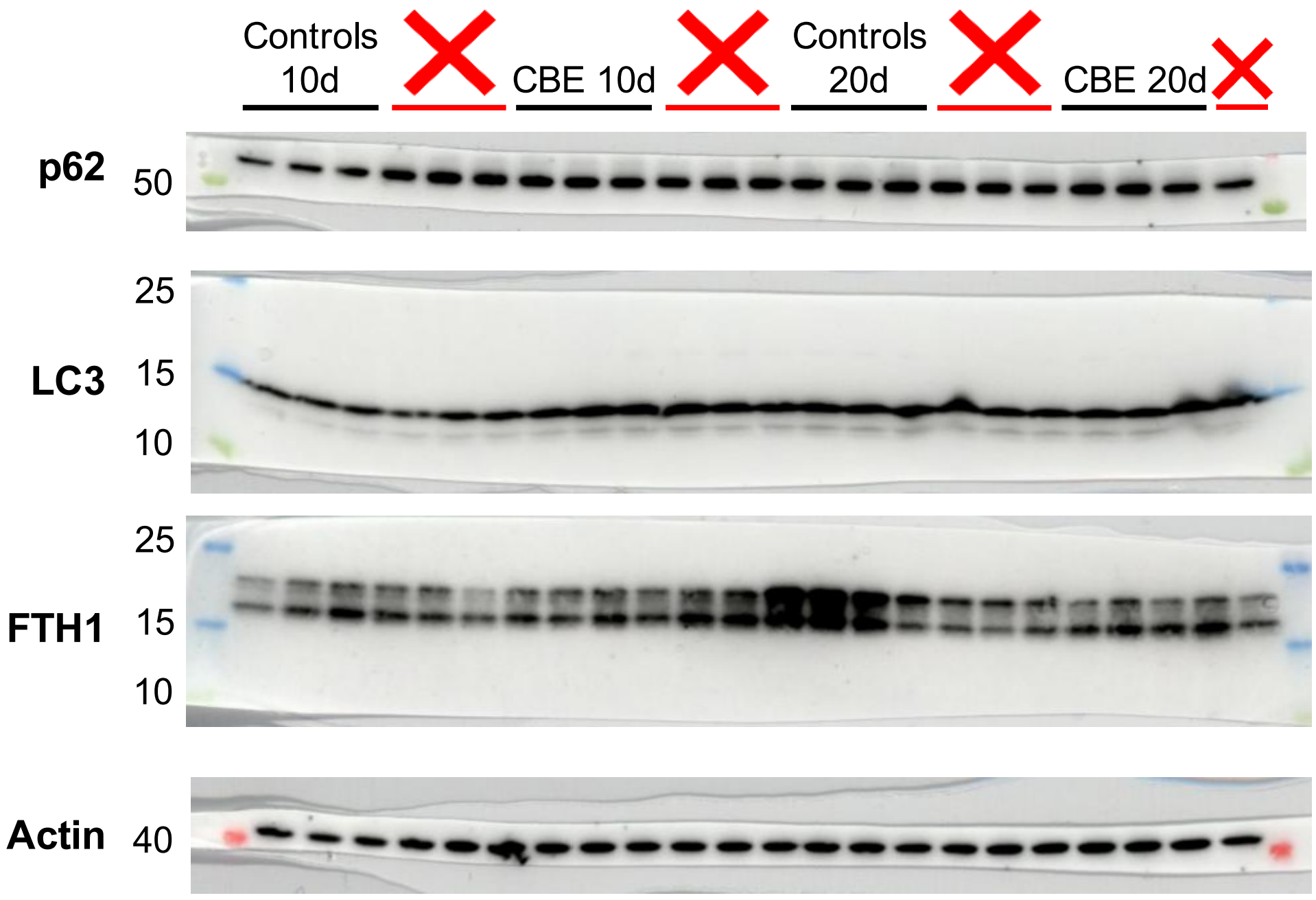

Supplemental Figure 4. Western-blots of p62, LC3 and FTH1 in CBE-treated cells *vs*. controls. Red crosses are samples that are not used in this article because these are other conditions not investigated in this study.

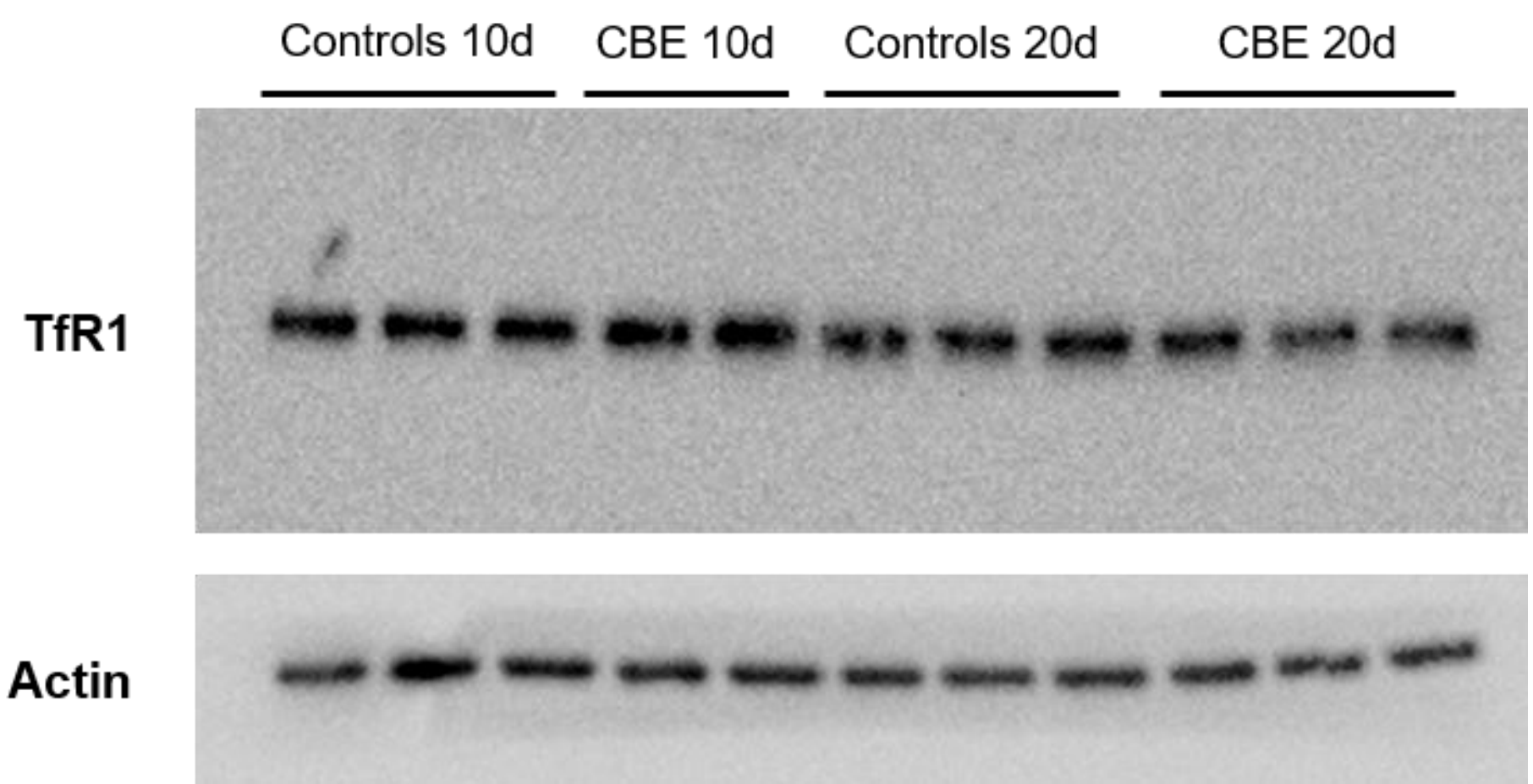

Supplemental Figure 5. Western-blots of TfR1 in CBE-treated cells *vs*. controls.

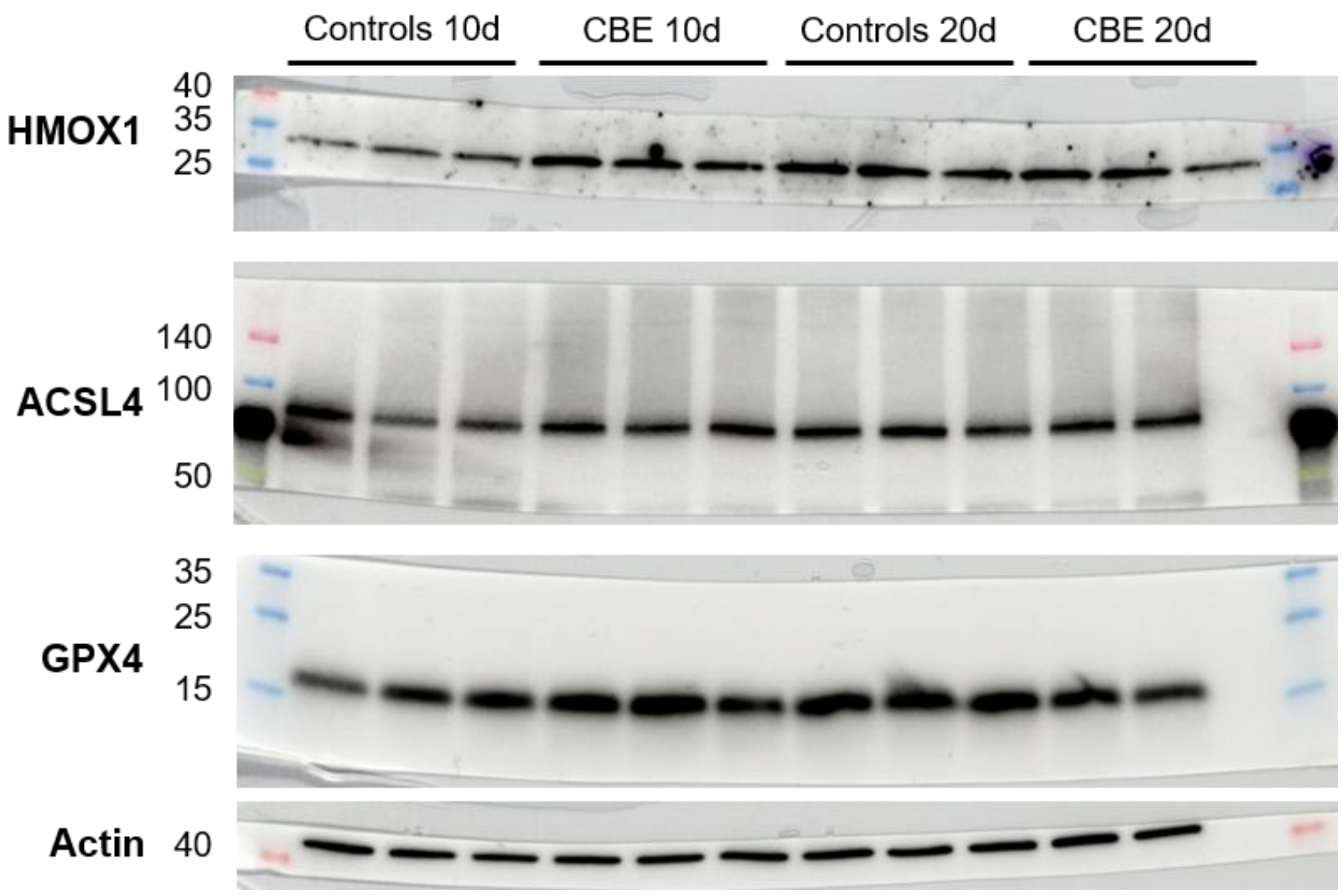

Supplemental Figure 6. Western-blots of HMOX1, ACSL4, GPX4 in CBE-treated cells *vs*. controls.

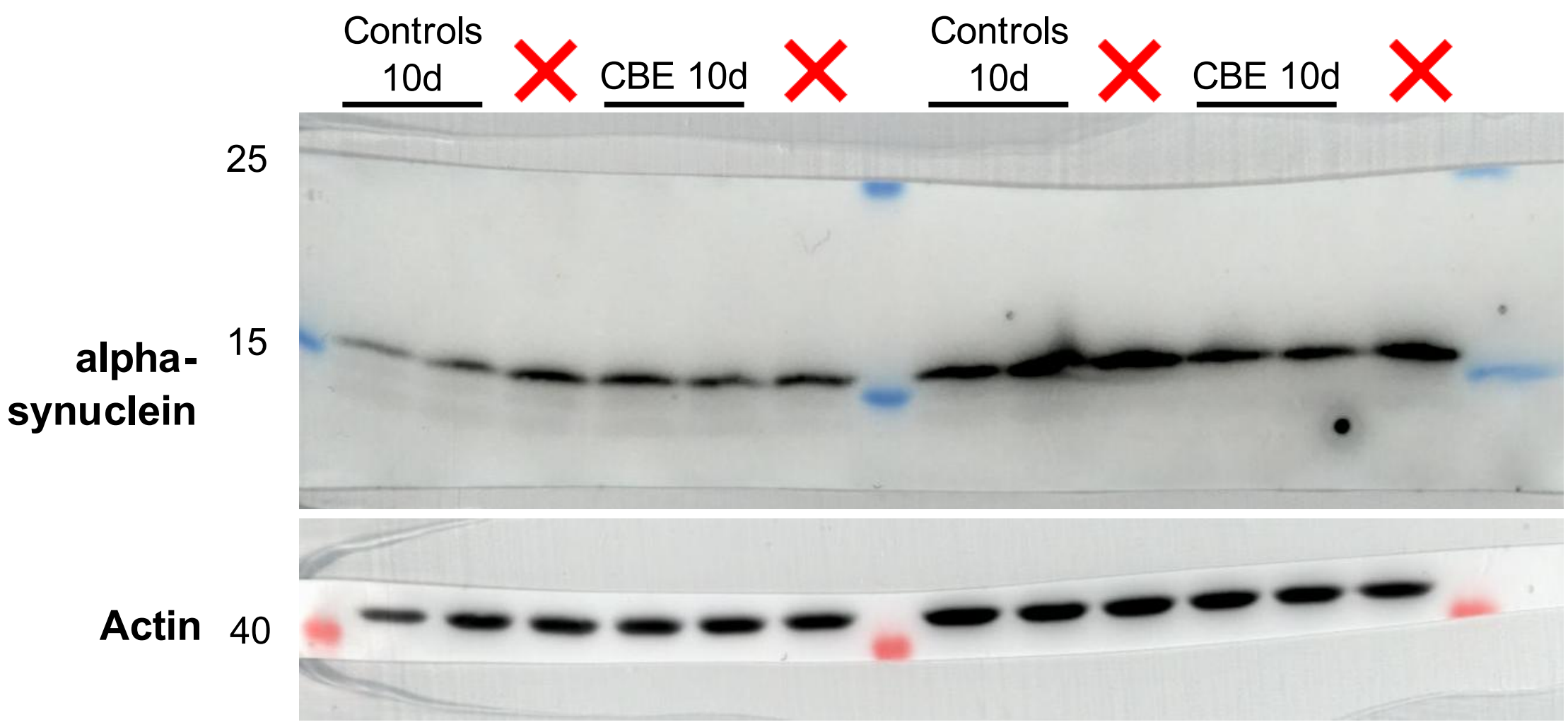

Supplemental Figure 7. Western-blots of alpha-synuclein in CBE-treated cells *vs*. controls. Red crosses are samples that are not used in this article because these are other conditions not investigated in this study.

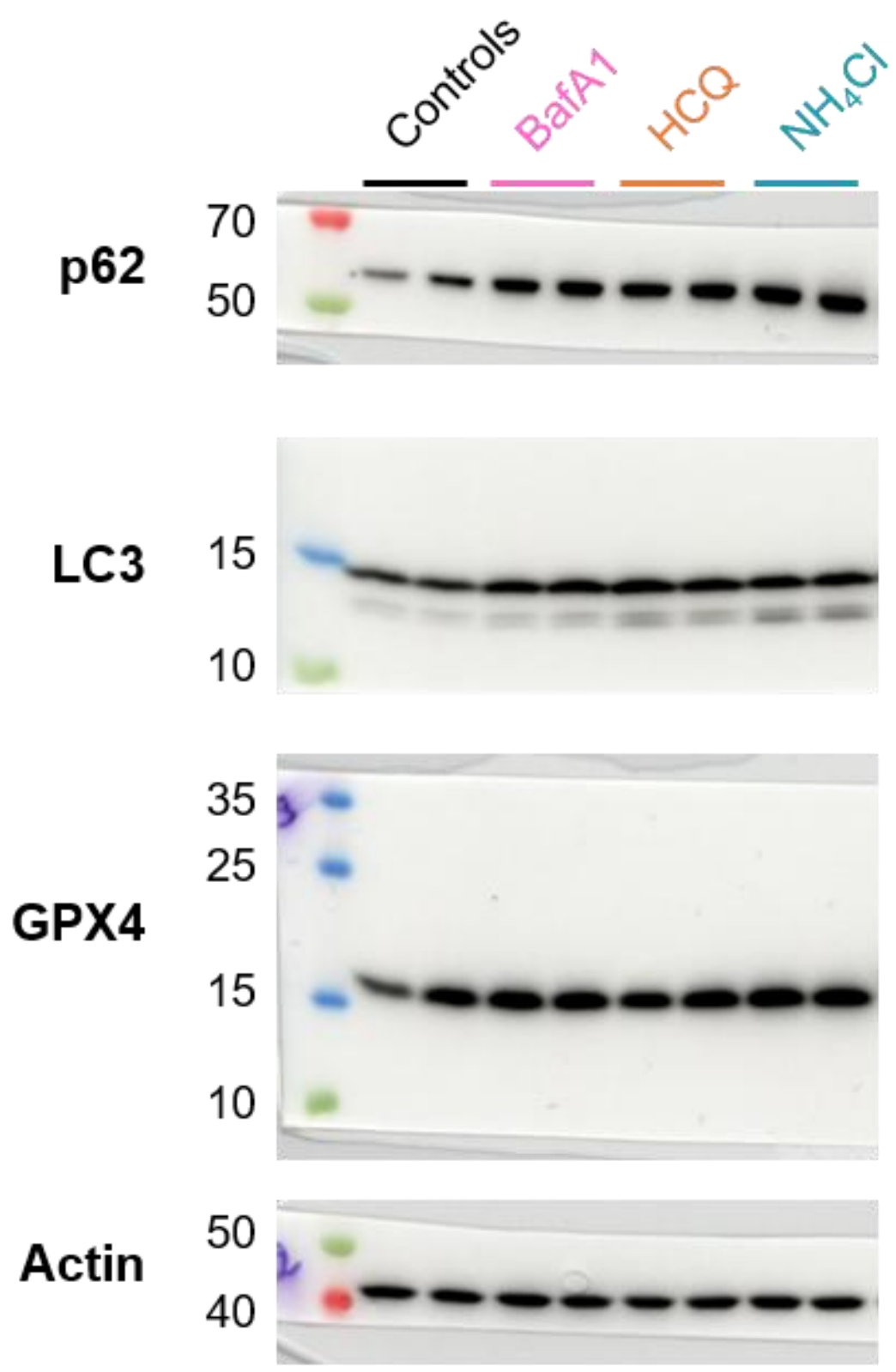

Supplemental Figure 8. Western-blots of p62, LC3 and GPX4 in cells exposed to inhibitors of autophagic flux.

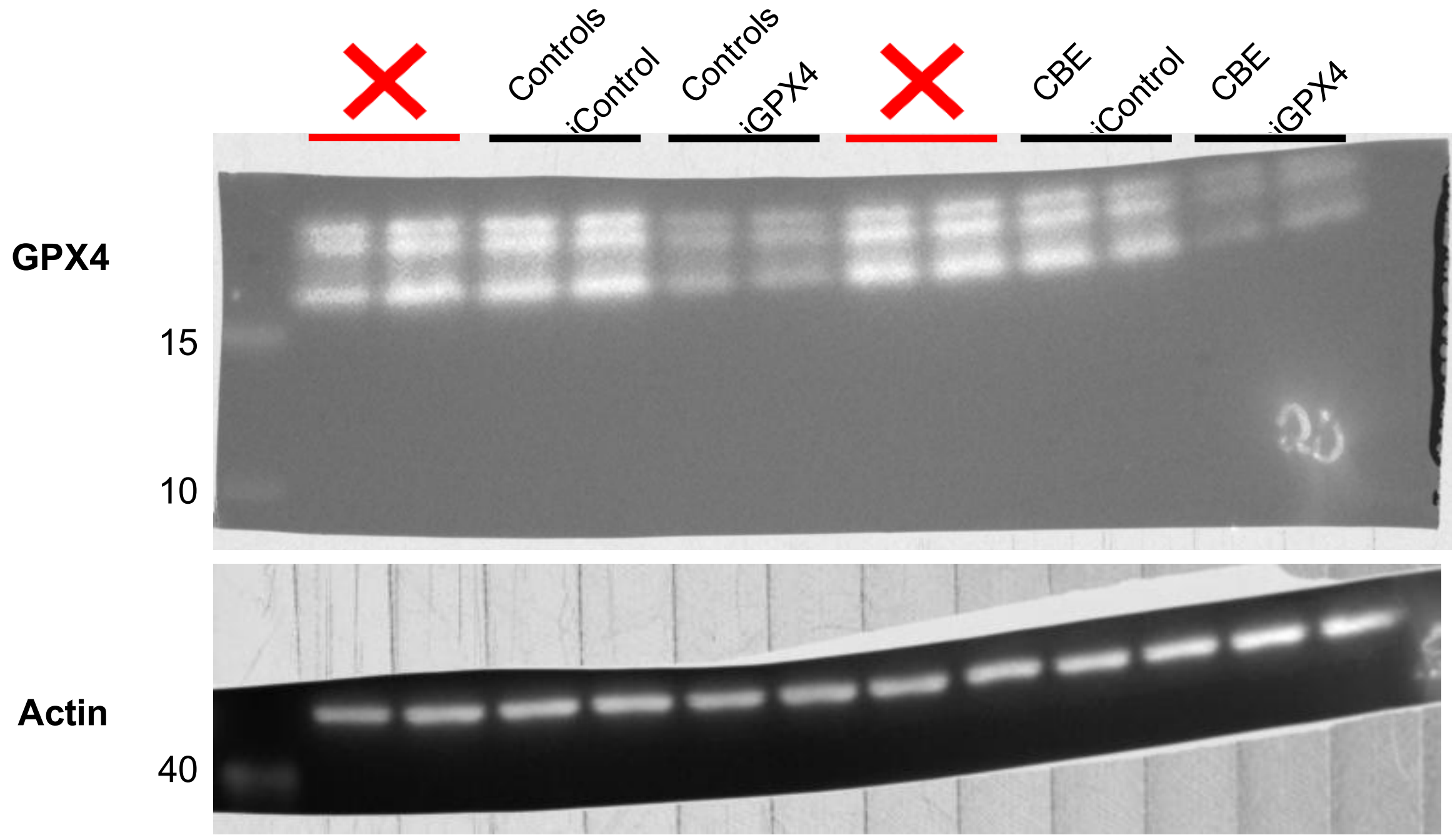

Supplemental Figure 9. Western-blots of GPX4 in cells transfected with siGPX4. Red crosses are samples that are not used in this article because these are other conditions not investigated in this study.
