# Supplementary material for: Long-term inhibition of lysosomal glucocerebrosidase activity promotes GPX4 stability and inhibits ferroptosis in dopaminergic neurons
Source: Cell Death Dis. 2026 Jun 8;17(1):717. doi: 10.1038/s41419-026-08833-8 (PMC13470012; doi:10.1038/s41419-026-08833-8)
Supplement: Supplementary file 6 [file 41419_2026_8833_MOESM6_ESM.pdf]

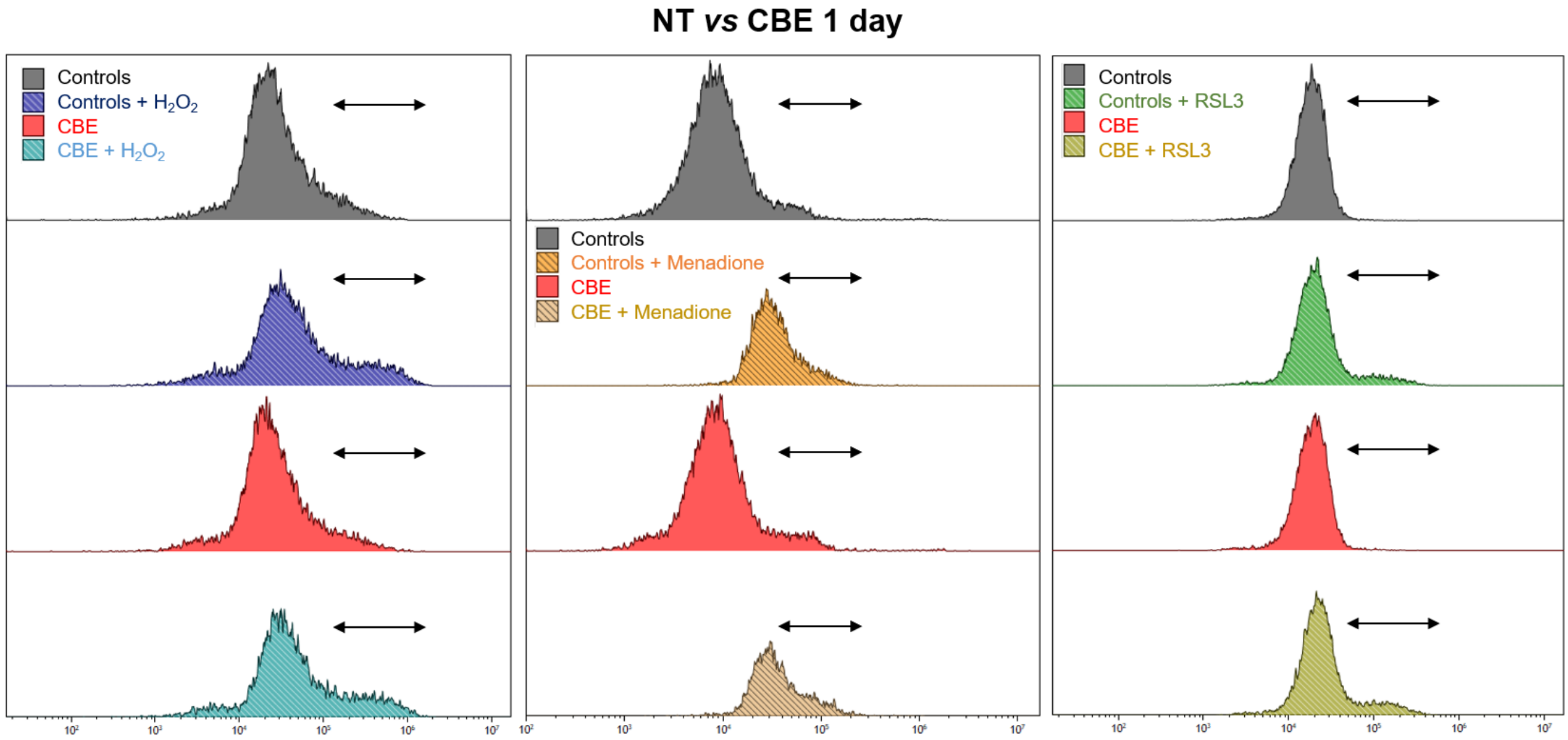

Supplemental Figure 10. Flow cytometry experiments in 1-day CBE and controls. LUHMES cells were treated with H_2O_2, menadione or RSL3 +/- CBE. Following the treatment, cells were stained with CM-H2DCFDA (left, cytosolic ROS), MitoSOX (middle, mitochondrial ROS) or C-11-BODIPY 581/591 (right, lipid ROS) probes. The arrows represent the measured parts.

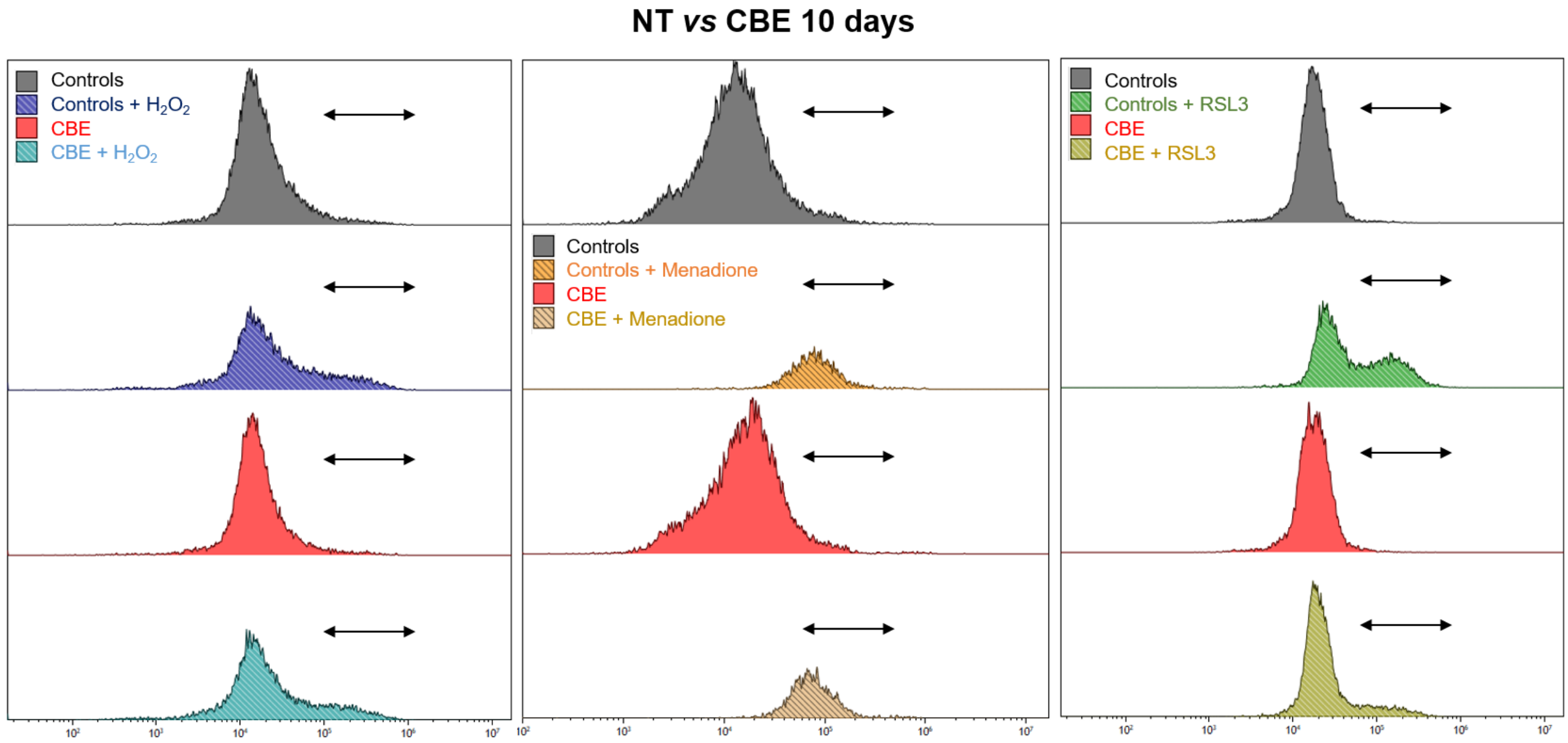

Supplemental Figure 11. Flow cytometry experiments in 10-day CBE and controls. LUHMES cells were treated with H_2O_2, menadione or RSL3 +/- CBE. Following the treatment, cells were stained with CM-H2DCFDA (left, cytosolic ROS), MitoSOX (middle, mitochondrial ROS) or C-11-BODIPY 581/591 (right, lipid ROS) probes. The arrows represent the measured parts.

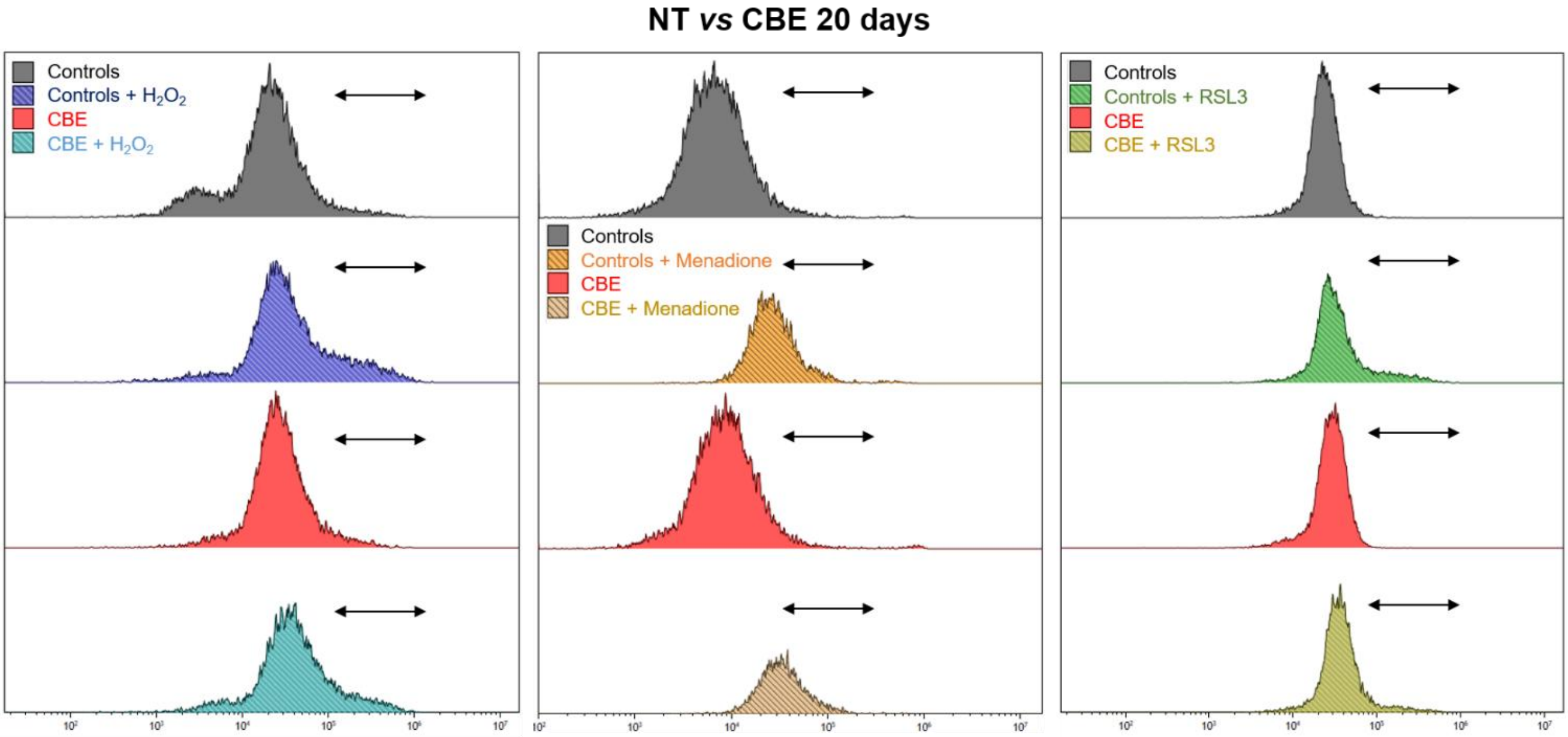

Supplemental Figure 12. Flow cytometry experiments in 20-day CBE and controls. LUHMES cells were treated with H_2O_2, menadione or RSL3 +/- CBE. Following the treatment, cells were stained with CM-H2DCFDA (left, cytosolic ROS), MitoSOX (middle, mitochondrial ROS) or C-11-BODIPY 581/591 (right, lipid ROS) probes. The arrows represent the measured parts.

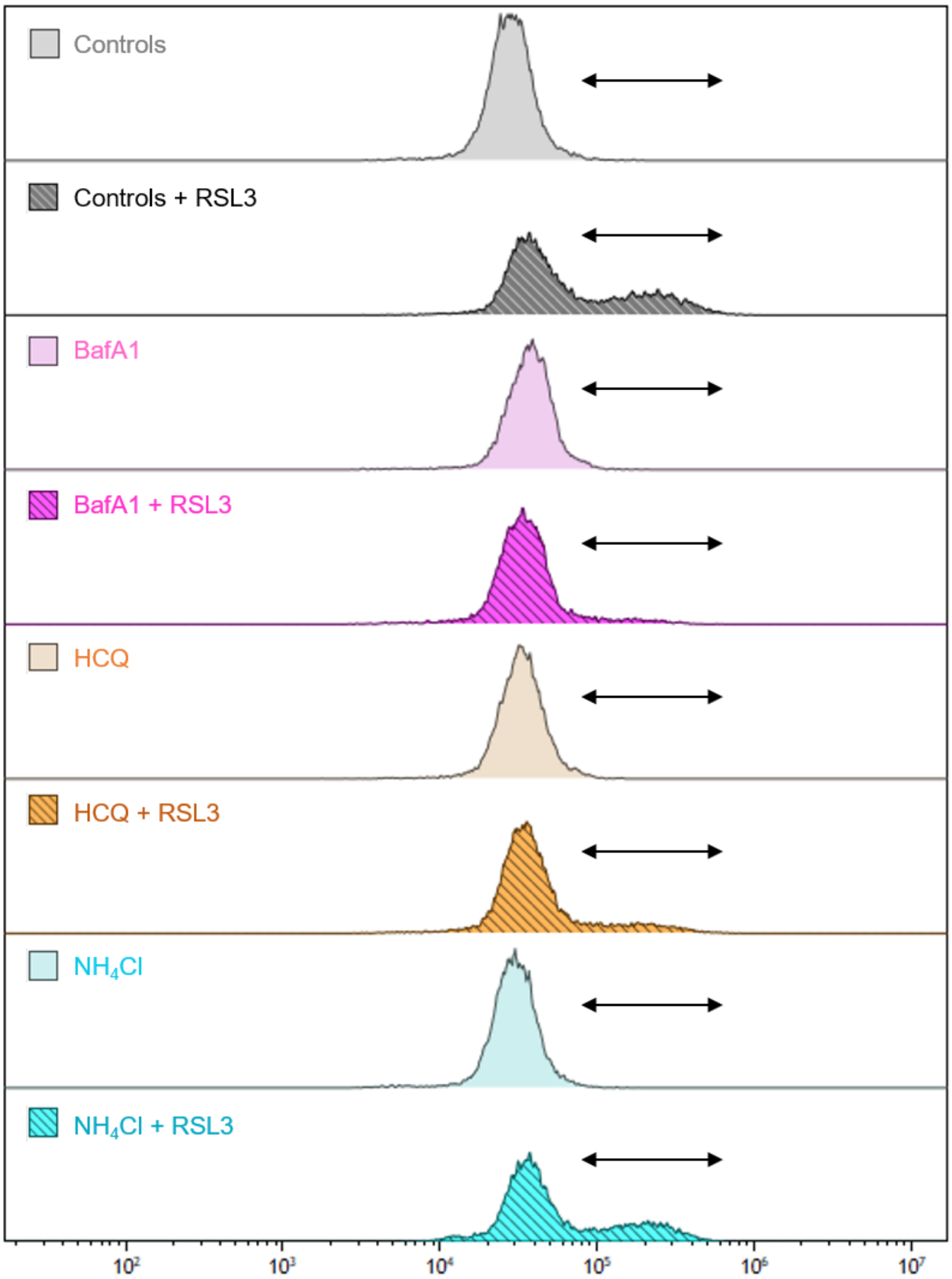

Supplemental Figure 13. Overlay of flow cytometry experiments in cells exposed to inhibitors of autophagic flux. LUHMES cells were pre-treated with BafA1, HCQ or NH_4Cl and were treated with RSL3 to induce lipid ROS. Following the treatment, cells were stained with C-11-BODIPY 581/591 probes to measure lipid ROS. The arrows represent the measured parts.
