# Supplementary material for: Long-term inhibition of lysosomal glucocerebrosidase activity promotes GPX4 stability and inhibits ferroptosis in dopaminergic neurons
Source: Cell Death Dis. 2026 Jun 8;17(1):717. doi: 10.1038/s41419-026-08833-8 (PMC13470012; doi:10.1038/s41419-026-08833-8)
Supplement: Supplementary file 7 [file 41419_2026_8833_MOESM7_ESM.pdf]

Supplemental Table 1. Primer sequences used for real-time PCR experiments.

Supplemental Table 2. Antibodies used for Western-blotting experiments.

Supplemental Figure 1. Sensitivity to ferroptosis is not mediated by ether-linked phosphatidylcholines.

Volcano plots of the changes in expression of PC species, with ePL colored in red for 10-day (A) or 20-day (B) CBE-treated cells *vs.* controls (four replicates were carried out per condition).

Supplemental Figure 2. 10-day CBE did not modify the alpha-synuclein protein levels.

Protein levels of alpha-synuclein in CBE-treated cells *vs*. controls.
